# Human AAA+ ATPase FIGNL1 suppresses RAD51-mediated ultra-fine bridge formation

**DOI:** 10.1093/nar/gkae263

**Published:** 2024-04-10

**Authors:** Kenichiro Matsuzaki, Akira Shinohara, Miki Shinohara

**Affiliations:** Department of Advanced Bioscience, Graduate School of Agriculture, Kindai University, Nara City, Nara 631-8505, Japan; Laboratory of Genome and Chromosome Functions, Institute for Protein Research, Osaka University, 3-2 Yamadaoka, Suita, Osaka 565-0871, Japan; Department of Advanced Bioscience, Graduate School of Agriculture, Kindai University, Nara City, Nara 631-8505, Japan; Agricultural Technology and Innovation Research Institute, Kindai University, Nara City, Nara 631-8505, Japan

## Abstract

RAD51 filament is crucial for the homology-dependent repair of DNA double-strand breaks and stalled DNA replication fork protection. Positive and negative regulators control RAD51 filament assembly and disassembly. RAD51 is vital for genome integrity but excessive accumulation of RAD51 on chromatin causes genome instability and growth defects. However, the detailed mechanism underlying RAD51 disassembly by negative regulators and the physiological consequence of abnormal RAD51 persistence remain largely unknown. Here, we report the role of the human AAA+ ATPase FIGNL1 in suppressing a novel type of RAD51-mediated genome instability. *FIGNL1* knockout human cells were defective in RAD51 dissociation after replication fork restart and accumulated ultra-fine chromosome bridges (UFBs), whose formation depends on RAD51 rather than replication fork stalling. FIGNL1 suppresses homologous recombination intermediate-like UFBs generated between sister chromatids at genomic loci with repeated sequences such as telomeres and centromeres. These data suggest that RAD51 persistence *per se* induces the formation of unresolved linkage between sister chromatids resulting in catastrophic genome instability. FIGNL1 facilitates post-replicative disassembly of RAD51 filament to suppress abnormal recombination intermediates and UFBs. These findings implicate FIGNL1 as a key factor required for active RAD51 removal after processing of stalled replication forks, which is essential to maintain genome stability.

## Graphical Abstract

**Graphical Abstract:**
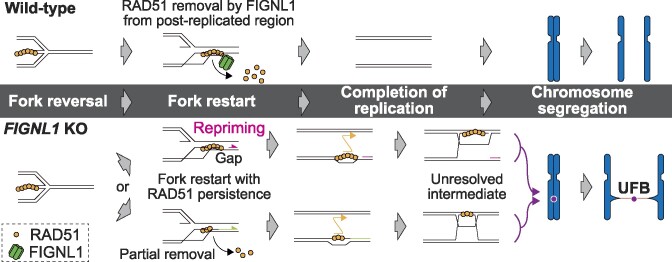

## Introduction

Maintaining genome stability during cell division ensures that daughter cells inherit an intact copy of the genetic information. Errors in the maintenance induce genome instability—a hallmark of cancer. The chromosome bridge, which is a linkage between chromosomes during anaphase, is an intermediate inducing genome instability by generating chromosomal breaks during segregation. The broken chromosome undergoes a further breakage-fusion-bridge (BFB) cycle, propagating catastrophic genetic information often associated with the formation of micro-nucleus (1,2). Several types of chromosome bridges have been reported. Anaphase bridges are generated by chromosome fusion and detected during anaphase using a DNA dye. Ultra-fine bridges (UFBs) are a type of anaphase bridge that cannot be detected through conventional DNA staining (3) but can be visualized by staining with antibodies against Plk1-interacting checkpoint helicase (PICH), Replication protein A (RPA), or Bloom helicase (BLM). Currently, five types of UFBs have been reported: fragile site-UFBs (FS-UFBs), centromeric-UFBs (C-UFBs), ribosomal-UFBs (R-UFBs), telomeric-UFBs (T-UFBs), and homologous recombination-UFBs (HR-UFBs) (4). FS-UFBs are produced in the region of incomplete replication as a linkage between sister chromatids (5). C-UFBs and R-UFBs are generated by dsDNA catenanes in the centromeric and ribosomal regions, respectively (6–8). T-UFBs are induced by telomere fusion or stalling of replication forks at the telomere region (9). HR-UFBs are induced by defects in the resolution of recombination intermediates (10). Both anaphase bridges and UFBs are resolved or cleaved at the exit of mitosis. Unresolved or non-cleaved bridges are detected as interphase chromosome bridges formed between interphase nuclei (11).

Homologous recombination (HR) is an error-free pathway to repair DNA double-strand breaks (DSBs). The DSB ends are processed by nucleases including the MRN (MRE11-RAD50-NBS1) complex, CtIP, EXO1 and DNA2 (with BLM-TOP3-RMI1/2) to produce 3′-overhanging single-stranded DNA (ssDNA) (12–14). RAD51 protein, which is a RecA homolog in eukaryotes, forms a nucleoprotein filament on ssDNA to catalyse homology search and strand invasion in HR (15,16). The RAD51-mediated recombination intermediates are dissolved by BLM or resolved by resolvases such as the SMX (SLX1-SLX4, MUS81-EME1, XPF-ERCC1) complex and GEN1 (17–19). In addition to HR, RAD51 plays a role in DNA replication. It protects nascent DNA strands at stalled replication forks from nucleolytic degradation by nucleases including MRE11, EXO1 and DNA2 (20–23) for maintaining the integrity of replication forks. However, uncontrolled accumulation of RAD51 on chromatin causes genome instability and growth defects during cell proliferation (24). RAD51 overexpression induces apoptosis in human cells and fruit flies and defects in chromosome segregation in fission yeast (24–26). Furthermore, high expression of RAD51 has been reported in various types of cancer (24). Nevertheless, the mechanism underlying RAD51-induced genomic instability remains ambiguous.

RAD51 filament assembly is regulated by two protein groups: RAD51 mediators that facilitate the recruitment of RAD51 and/or stabilize RAD51 filaments, and RAD51-dismantling enzymes, also known as anti-recombinases, that dissociate RAD51 from DNAs. RAD51-dismantling enzymes are highly conserved among species. Multiple RAD51-dismantling enzymes have been isolated in single species, implying that the inhibition of inappropriate RAD51 assembly is essential for cell proliferation. In humans, BLM, PARI, FBH1, RECQL5 and FIGNL1 disassemble RAD51 filaments, and their dysfunction is often associated with increased genomic instability (27–31). Although each RAD51-dismantling enzyme is critical for maintaining genome stability, their functional distinction remains unclear.

One of the most enigmatic anti-recombinases is FIGNL1, which is an AAA+ ATPase that interacts with several DSB repair proteins such as RAD51, SWSAP1, SPIDR and FLIP(FIRRM) (29,32–34). FIGNL1 disrupts RAD51 filaments by facilitating RAD51 ATP activity (29). FIGNL1 binds directly to RAD51 through its conserved FxxA motif (29,32). Unlike other anti-recombinases, FIGNL1 does not have a putative helicase motif, and a purified FIGNL1 protein with FxxA and ATPase domains does not bind to DNA (29). FIGNL1 reportedly disrupts RAD51 filaments via a mechanism distinct from that of other helicases and is possibly involved in HR after RAD51 filament formation (32). Nevertheless, the physiological conditions under which FIGNL1 disrupts RAD51 filaments remain unclear.

In this study, we report that the deletion of the *FIGNL1* gene in human cells reduced proliferation, increased spontaneous RAD51-focus formation, and induced spontaneous DNA damage. In *FIGNL1* knockout (KO) cells, RAD51 was normally recruited to the stalled replication fork but did not dissociate after fork restart. Importantly, the deletion of *FIGNL1* significantly increased UFB formation. Strikingly, we show that the inhibition of RAD51-filament formation reduced UFB formation and DNA damage. Our results suggest that FIGNL1 facilitates RAD51 disassembly from post-replicative regions to prevent UFB formation, which is caused by abnormal recombination intermediates. Our findings revealed the risk associated with improper persistence of RAD51 and the necessity of the RAD51-dismantling enzyme in the maintenance of genome stability.

## Materials and methods

### Cell lines

U2OS cells (ATCC HTB-96), HeLa (ATCC CCL-2), and their derivative cell lines were maintained in DMEM supplemented with 10% FBS and antibiotics. Cells expressing Myc-FIGNL1, Myc-FIGNL1-EE or FLAG-FIGNL1 were generated by transfection of U2OS cells with pIRES-Puro3-Myc-FIGNL1 and pIRES-Puro3-Myc-FIGNL1-EE.

### Antibodies

Anti-γH2AX (1:500 Millipore 05-636), anti-RAD51 (1:500 Millipore ABE257), anti-PICH (1:50 Millipore 04-1540), anti-FANCD2 (1:500 Novus Biologicals NB100-182), anti-PCNA (1:500 Santa Cruz sc56), anti-centromere (1:400 ImmunoVision hct-0100), anti-rabbit Alexa Fluor 488 (1:500 Invitrogen A11034), anti-mouse Alexa Fluor 488 (1:500 Invitrogen A11029), anti-mouse Alexa Fluor 594 (1:500 Invitrogen A11032), and anti-human Alexa Fluor 488 (1:500 Invitrogen A11013) antibodies were used for immunofluorescence analysis. Anti-PCNA (1:200 Santa Cruz sc56), anti-RAD51 (1:500 Millipore ABE257), anti-histone H3 (1:500 Abcam ab1791 or 1:500 Abcam ab10799), anti-Myc (1:3000 Nacalai tesque 04362–34), anti-HA (1:2000 Covance MMS-101R), anti-tubulin (Sigma, T4026), anti-FLAG(1:3000 Wako 012-22384), anti-GFP (1:5000 Abcam ab290) anti-mouse AP-conjugated (1:5000 Promega S3721), and anti-rabbit AP-conjugated rabbit (1:5000 Promega S3738) antibodies were used for western blotting analysis. Anti-BrdU (1:50 or 1:25, BD 347580) antibody was used for DNA combing.

### Generation of *FIGNL1* KO cells using CRISPR/Cas9 system

*FIGNL1* KO cells were generated using Guide-it sgRNA In Vitro Transcription Kit (Takara Bio 632635) and recombinant Cas9 (Takara Bio 632641) according to the manufacturer's protocols. Templates for sgRNA were generated using PCR with sgRNA scaffold template and primer containing sgRNA target sequence. The PCR products were used for the *in vitro* transcription reaction. Two sgRNA targeting human *FIGNL1* genes were purified and used for the electroporation with recombinant Cas9. Transfected U2OS and HeLa cells were seeded in 10-cm dishes. After electroporation for 10 days, 48 colonies were isolated, propagated, and subjected to genotyping.

### Primers for sgRNA template

hFIGNL1 Guide-it FW primer1:

CCTCTAATACGACTCACTATAGGTGGCATATGTACC-

GGACCGAGTTTAAGAGCTATGC

hFIGNL1 guide-it FW primer2:

CCTCTAATACGACTCACTATAGGTCGAACTTGATCC-

GGTGTTAGTTTAAGAGCTATGC

hFIGNL1 genotyping primers for PCR and sequencing

hFIGNL1KO-check-f2: ACAGTACCTGGAGTGAAACTGCTTGTGTTC

hFIGNL1KO-check-r2: TCCTGTCCTATATGCGCTCTACCAGATGAC

### Measurement of cell proliferation

U2OS cells were seeded at a density of 2 × 10^4^ cells per well in a 6-well plate and counted every 2 days using a Cell counter model R1 (Olympus).

Cell viability was assessed using CellTiter-Glo (Promega G7570) according to the manufacturer's protocol. This reagent determines the number of viable cells in the culture by producing a luminescent signal proportional to the ATP content. Chemiluminescence was measured using a Chameleon luminometer (Hidex). For the analysis of *FIGNL1* KO cells, U2OS cells were seeded at a density of 1000 cells/well in 96-well plates and analysed 6 days after seeding. For the analysis of RAD51 inhibitors, U2OS cells were seeded at a density of 1000 cells/well in 96-well plates. The relative luminescence to control cells or untreated cells was calculated. After 24 h of incubation, the indicated concentration of B02 was added. The cells were incubated for 7 days and analysed. For the analysis of HU, U2OS cells were seeded at a density of 1000 cells/well in a 96-well plate and incubated in the media containing the indicated concentration of HU for 24 h. After HU treatment, the cells were washed and incubated in HU-free media for 5 days.

### Clonogenic survival

U2OS cells were plated in triplicate on 10-cm dishes. After 7 days, the cells were fixed and stained in 4% crystal violet in 20% ethanol. The number of colonies was counted and normalized for plating efficiency. Sensitivity to camptothecin was assessed 24 h after seeding by treating the U2OS cells with the indicated concentration of camptothecin for 22 h.

### Immunofluorescence staining

To detect RAD51, γH2AX, centromere, and PCNA, cells were cultured on coverslips and permeabilized with CSK buffer (10 mM PIPES at pH 6.8, 100 mM NaCl, 300 mM sucrose, 3 mM MgCl_2_, 1 mM EGTA, 0.5% Triton X-100, 1× protease inhibitor cocktail (Roche 11873580001), and 1× PhosSTOP (Roche 4906837001)) for 5 min on ice. After washing in PBS, the cells were fixed with 2% PFA (Sigma) for 15 min at room temperature. The coverslips were blocked in PBST containing either 3% BSA or 10% goat serum and 5% BSA for 30 min at room temperature. The cells were incubated overnight at 4°C with the primary antibodies. The coverslips were washed thrice with PBST and incubated with secondary antibodies for 1 h at room temperature. After washing with PBST, the coverslips were mounted with Vectashield medium (Vector Laboratories H-1000).

To analyse CPT-induced RAD51 foci, cells were treated with 1 μM CPT for 1 h. Cells were washed and cultured in CPT-free medium for 8, 24, 48 and 72 h before being fixed.

For the detection of histone H3 pS10, anaphase bridges and UFBs, the cells cultured on coverslips were washed with PBS and fixed with PFA solution (4% PFA, 20 mM PIPES at pH 6.8, 1 mM MgCl_2_, 10 mM EDTA and 0.2% Triton X-100).

### EdU labelling

EdU incorporation and detection for microscopic analysis were performed using the Click-iT EdU Cell Proliferation kit (Invitrogen C10339) according to the manufacturer's protocol. U2OS cells were treated with 20 μM EdU for 10 min at 37°C.

### DNA combing

DNA combing was performed according to a previously described method with minor modification (35–37). U2OS cells were pulse-labelled with 20 μM IdU for 30 min at 37°C, washed, and incubated in the presence of 2 mM HU for 4 h at 37°C. After HU treatment, the cells were washed and pulse-labelled with 100 μM EdU for 30 min at 37°C to monitor fork restart. The cells were resuspended in PBS, mixed with 1% melted agarose at a 1:1 ratio, and poured into a plug mould, which was incubated in DNA combing lysis buffer (400 mM EDTA, 1% *N*-lauroyl-sarcosine, 1 mg/ml proteinase K) for 18 h at 50°C and washed twice with 0.5 M EDTA and thrice with TE. A single plug was melted for 30 min at 65°C and treated with β-agarase (NEB M0392) for 24 h at 42°C. Genomic DNA extracted from the agarose plug was diluted with 150 mM MES (pH 5.5) and stretched on an APS-coated glass slide (MATSUNAMI APS-01). The slides were baked for 24 h at 60°C, denatured in 2× SSC/50% formamide for 13 min at 73°C, dehydrated by sequential immersion in 70%, 90% and 100% EtOH for 2 min, and air-dried. EdU-incorporated DNA strands were visualized using a Click-iT EdU Cell Proliferation kit according to the manufacturer's protocol. Subsequently, the IdU-labeled DNA strands were stained with an anti-BrdU antibody for 30 min at room temperature and an anti-mouse Alexa 488 antibody for 30 min at room temperature. The slides were mounted with Vectashield medium and analysed under a microscope.

### S1 fibre assay

S1 fibre assay was performed as previously described (38,39). Cells were sequentially labeled with 20 μM IdU for 20 min, followed by 200 μM CldU for 40 min. Labelled cells were permeabilized with CSK100 buffer (100 mM NaCl, 10 mM MOPS (pH 7.3), 3 mM MgCl_2_, 300 mM sucrose, 0.5% TX-100) for 10 min at room temperature. After washing with S1 nuclease buffer (30 mM NaOAc, 10 mM Zn(OAc)_2_, 5% glycerol, 50 mM NaCl, pH 4.6), samples were treated with 10 U/ml S1 nuclease (ThermoFisher 18001016) for 30 min at 37°C. Cells were harvested using a cell scraper and fixed in a fixative solution (MeOH: AcOH = 3:1). Cells were spotted onto the glass slides and dried for 3 min at room temperature. Slides were immersed in lysis solution (200 mM Tris–HCl (pH 7.5), 50 mM EDTA, 0.5% SDS) for 20 min at 37°C and genomic DNA was stretched by tilting glass slides. DNA fibres were denatured in 2.5 M HCl for 1 h. After washing slides with PBS, slides were blocked in PBS containing 5% BSA and 0.1% Tween-20 for 1 h and incubated with anti-BrdU antibody (BD Bioscience 347580, 1:25 and Abcam ab6326, 1:100) for 2 h at 37°C. After washing with PBS containing 0.1% Tween-20, slides were incubated with Alexa Fluor 488-conjugated anti-rat IgG (Invitrogen A11006) and Alexa Fluor 594-conjugated anti-mouse IgG (Invitrogen A11032) for 1 h. The stained slides were washed with PBS containing 0.1% Tween-20 and mounted with VectaShield.

### iPOND

The iPOND assay was performed as previously described (37,40). For the analysis of HU-treated cells, U2OS cells were incubated in media containing 10 μM EdU for 10 min or media containing 10 μM EdU and 2 mM HU for 4 h. For thymidine chase experiments, HU-treated cells were washed and incubated in media containing 10 μM thymidine for 10 min or 60 min. For iPOND assay in an unchallenged condition (Supplementary Figure S3B), U2OS cells were incubated in media containing 10 μM EdU for 30 min. Subsequently, cells were washed and incubated in media containing 10 μM thymidine for 60 min. The EdU-labeled cells were fixed in 1% formaldehyde for 20 min and quenched by the addition of 125 mM glycine. The cells were washed in PBS, permeabilized in a permeabilization buffer (0.25% Triton X-100/PBS) for 30 min at room temperature, washed in PBS, and subjected to a click reaction. The cells were lysed in a lysis buffer (50 mM Tris–HCl [pH 8.0] and 1% SDS) and sonicated using Bioruptor. Then, 15 μl of the sonicated samples were saved as input. Proteins bound to EdU-labelled DNA were precipitated from cell lysates using streptavidin beads and eluted with SDS buffer (120 mM Tris–HCl [pH 6.8], 4% SDS, 0.2 M DTT, 10% glycerol, and 0.04% Bromophenol blue). The eluted samples and inputs were subjected to western blotting.

### Cell synchronization

Cell synchronization was performed as previously described (41). U2OS cells were seeded on coverslips for the analysis of UFBs and anaphase bridges or on a 10-cm dish for metaphase spreads. Twenty-four hours after seeding, the cells were treated with 2 mM thymidine for 18 h. The cells were washed and incubated in DMEM containing 10% FBS for 9 h. The cells were then incubated in 2 mM thymidine-containing media for 17 h. After washing, the cells were released into thymidine-free media for 7 h and treated with 9 μM RO-3306 (Selleckchem S7747) for 12 h. To detect UFBs and anaphase bridges, the cells were incubated in RO-3306-free media for 1 h and fixed with 4% PFA. For the analysis of metaphase spreads, the cells were incubated in media containing 0.2 μg/ml colcemid (Gibco 15212-012) for 40 min and fixed with methanol/acetate solution.

### Plasmid transfection

U2OS or 293T cells were transfected with the indicated plasmid using XtremeGENE HP transfection reagent (Roche, 6366236001) according to the manufacturer's protocol. U2OS cells were transfected using 5 μg of plasmid DNA and 15 μl of XtremeGENE HP on a 10-cm dish. After transfection for 24 h, the medium was replaced with DMEM (Gibco) containing 10% FBS. At 72 h after transfection, a cell scraper was used to harvest the 293T cells, which were subjected to immunoprecipitation and western blotting. Stable cell lines were generated by seeding the cells in a 10-cm dish 24 h after transfection and selecting 250 ng/ml of puromycin for 7 days. Finally, 48 colonies were picked, propagated, and subjected to western blotting to verify protein expression.

To express FLAG-GEN1, cells were transfected with pIRES-Puro3-FLAG-GEN1 using XtremeGENE HP and incubated for 3 h in thymidine-free media subsequent to the initial thymidine treatment.

### Inhibition of RAD51

RAD51 inhibitor B02 was purchased from Cayman Chemical Company (22133). Immunofluorescence staining of RAD51 and γH2AX was performed by treating the cells with 1 μM or 5 μM B02 for 48 h before fixing. For cell synchronization and analysis of UFBs, B02 was added to the medium after the first round of thymidine blocking. The cells were incubated in the presence of 1 μM B02 until RO-3306 was washed out.

### Immunoprecipitation (IP)

IP experiments were performed as previously described (29). Cells were lysed in 500 μl of benzonase buffer (20 mM Tris–HCl [pH 7.5], 40 mM NaCl, 2 mM MgCl_2_, 0.5% NP-40, 50 U/ml benzonase (Millipore 70746), 1× Protease inhibitor cocktail, and 1× PhosSTOP) for 10 min at 4°C. NaCl was added to a final concentration of 500 mM. After 30 min of incubation, centrifugation was performed to remove the cells. The resultant whole cell extracts (WCEs) were diluted 1:3 with No-salt IP buffer (25 mM Tris–HCl [pH 7.5], 1.5 mM DTT, 15% glycerol, 1× protease inhibitor cocktail, and 1× PhosSTOP). Then, 12 μl of EZview Red anti-Myc affinity Gel (Millipore E6654) or anti-FLAG beads (Wako 012–22781) was added to 300 μl of the diluted extracts and incubated for 90 min at 4°C, followed by washing of the beads in IP buffer (25 mM Tris–HCl [pH7.5], 150 mM NaCl, 1.5 mM DTT, 10% glycerol, 0.25% NP-40, 1× protease inhibitor cocktail, and 1× PhosSTOP). The immunoprecipitated proteins were eluted with 50 μl of sample buffer (120 mM Tris–HCl [pH 6.8], 4% SDS, 10% β-mercaptoethanol, 10% glycerol and 0.04% Bromophenol blue).

### Western blotting

The IP and WCE samples were separated using 10–20% SuperSep Ace (Wako 198-15041) and transferred onto a PVDF membrane (Millipore IPVH00010). The membranes were blocked using 5% skim milk in TBST for 30 min and initially incubated overnight at 4°C with the primary antibody and then for 30 min with the secondary antibody. The proteins were detected using a BCIP-NBT alkaline phosphatase solution (Nacalai, 03937-60). The images were cropped and processed using Photoshop 2020 (Adobe, USA). Uncropped images were shown in Supplementary Figure S11. The signal intensity of each band was quantified using ImageJ software and relative intensities to untreated wild-type control were calculated.

### IF-FISH

Cells on coverslips were fixed and stained as described for ‘Immunofluorescence staining.’ After incubation with the secondary antibody, the cells were washed thrice in PBS and fixed with 2% PFA for 5 min. After washing with PBS, the cells were consecutively dehydrated in 70%, 95% and 100% ethanol and air-dried, followed by hybridization with a PNA probe (Biologica F1006) in a hybridizing solution (70% formamide, 0.5% blocking reagent (Roche 11096176001), 10 mM Tris–HCl [pH 7.2]) for 12 h at 4 ºC. The coverslips were washed twice with a washing solution (70% formamide and 10 mM Tris–HCl [pH 7.2]) and thrice with PBS. After washing, the coverslips were stained with DAPI and mounted with Vectashield medium.

### Telomere and centromere metaphase FISH

Metaphase spreads were prepared according to a standard protocol (29,36). Briefly, cells were synchronized using a double thymidine block and released into thymidine-free media. After incubation for 7 h, 9 μM RO-3306 was added, and the cells were incubated at 37°C for 12 h. After washing twice with media, the cells were incubated in media containing 0.1 μg/ml colcemid for 30 min. The cells were collected through mitotic shake-off, washed with PBS, and resuspended in a 75 mM KCl solution. After incubation for 20 min, the fixation solution (MeOH:AcOH; 3:1) was added dropwise, and the cells were incubated at 4°C for 5 min, followed by washing and resuspension in the fixation solution. The cell suspension was dropped on a glass slide, washed with the fixation solution, and a hybridizing solution (70% formamide, 0.5% blocking reagent, 10 mM Tris–HCl [pH 7.2] with 17.7 nM TelG-Cy3 probe (Biologica F1006) or 50 nM CENPB-Alexa 488 (PNA Bio, F3005)) was added to the slides. The samples were denatured at 75°C for 7 min. After overnight incubation, the slides were washed twice in washing solution (70% formamide and 10 mM Tris–HCl [pH 7.2]) and thrice in PBS. During the second wash, the slides were washed in PBS containing DAPI to visualize metaphase chromosomes. After washing, the slides were mounted with Vectashield.

### Centromere CO-FISH

Metaphase spreads were prepared as described for ‘Telomere metaphase FISH.’ Media containing 7.5 μM BrdU and 2.5 μM BrdC were used after the second block until mitotic shake-off. The slides were treated with 0.5 mg/ml RNase A at 37°C for 30 min and 0.5 μg/ml Hoechst at 25°C for 15 min, exposed to 365 nm UV light at 6500 J/m^2^ in a UV cross-linker, and treated with 10 U/μl Exonuclease III (Promega M1811) at 37°C for 30 min. After washing with PBS, the slides were dehydrated through sequential immersion in 70%, 90%, and 100% EtOH for 5 min and air-dried. Then, the slides were incubated in a hybridizing solution containing a 50 nM CENPBR-Cy3 (PNA Bio F3009) probe at 25°C for 2 h. After washing with the Hybridization wash1 (70% formamide and 10 mM Tris–HCl [pH 7.2]), the slides were incubated in a hybridizing solution containing 100 nM CENPB-Alexa 488 (PNA Bio F3005) at 25°C for 2 h. The slides were washed once with Hybridization wash1 for 15 min and thrice with Hybridization wash2 (100 mM Tris-HCl [pH 7.2], 150 mM NaCl, 0.08% Tween-20) for 5 min. The metaphase chromosomes were stained with Hybridization wash2 containing DAPI, dehydrated through sequential immersion in 70%, 90% and 100% EtOH for 5 min, and mounted using ProLong Gold (Invitrogen P10144).

### siRNA

After the first round of thymidine treatment, the U2OS cells were released, incubated in thymidine-free media for 3 h, and transfected with siRNA using RNAiMAX transfection reagent (Invitrogen 13778150) for 6 h. Then, 2 mM thymidine was added, and cells were incubated for 17 h. After washing, the cells were incubated in thymidine-free media for 7 h, treated with 9 μM of RO-3306 for 12 h, and washed and fixed as described for ‘immunofluorescence staining.’ For the analysis of cells in the S/G2 phase, the cells were fixed before RO-3306 treatment (S/G2 sample). To analyse the cells in the G1 phase, they were treated with RO-3306, incubated with 2 mM thymidine for 10 h, and fixed (G1 sample).

siControl: UAGCGACUAAACACAUCAA

siFIGNL1: GUGCACAGAUAUUACGCAU

siGEN1: GUAAAGACCUGCAAUGUUA

siMUS81: CAGCCCUGGUGGAUCGAUA

### RT-qPCR

Total RNA from U2OS cells was isolated using the RNeasy Mini kit (Qiagen 74106) according to the manufacturer's protocol. The expression of FIGNL1 and ACTB were assessed using the Luna Universal One-step RT-qPCR kit (NEB E3005).

### Primers for RT-qPCR

hFIGNL1-qPCR-f: GGAGCAACAAATCGGCCACAA

hFIGNL-1qPCR-r: ATGTCTGCTCCTGAAAACGCATC

hACTB-qPCR-f: CGTGCGTGACATTAAGGAGAAG

hACTB-qPCR-r: GGAAGGAAGGCTGGAAGAGTG

### Statistical analysis

GraphPad Prism 10 was used for statistical analysis. Unpaired *t*-test was used for the quantifications of cells with RAD51 foci, cells with γH2AX foci, restarted forks, chromatin bridges, UFBs, H3-pS10-positive cells, EdU-incorporated cells, FIGNL1 mRNA, MN formation, and cell viability. The Mann–Whitney *U*-test was used for focus counting, measurement of DNA fibre length treated with S1 nuclease and metaphase spread experiments.

## Results

### Function of *FIGNL1* in maintaining genome stability

**Figure 1.**
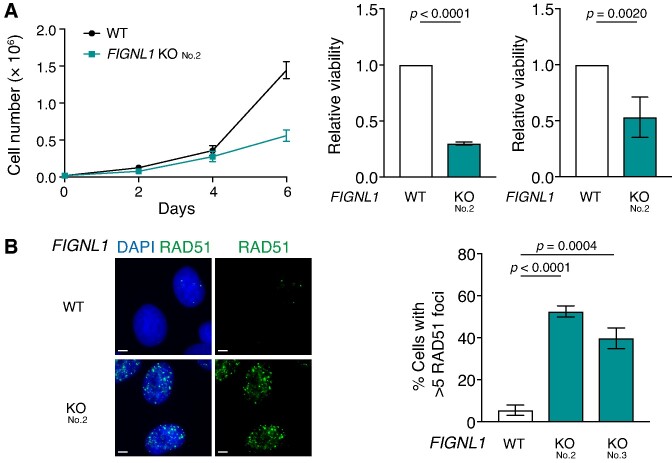
Impaired cell proliferation and accumulation of RAD51 in *FIGNL1* KO cells. (**A**) Left, growth curve of control U2OS and *FIGNL1* KO No.2 (Supplementary Figure S1) cells. Relative viability from clonogenic assay (middle) and ATP-based cell viability assay (right) of control U2OS and *FIGNL1* KO cells. Relative numbers to control U2OS cells are shown. Data are presented as mean ± s.d. (*n* = 4, all biologically independent). (**B**) Left, Representative images of immunofluorescence analysis of RAD51 foci in control U2OS cells (top) and *FIGNL1* KO cells (bottom). Scale bar = 5 μm. Right, Quantification of RAD51 focus-positive cells (>5 foci/cell) in the indicated cell lines. More than 200 cells were counted for each sample. Data are presented as mean ± s.d. (*n* = 3, all biologically independent).

To investigate the function of *FIGNL1* in the maintenance of genome stability, we generated *FIGNL1* knockout (KO) cell lines using the CRISPR/Cas9 system. The Human *FIGNL1* gene has two exons with the coding region in the second exon (Supplementary Figure S1A, B). Therefore, we designed two sgRNAs for CRISPR/Cas9-mediated gene editing to delete the second exon in the U2OS cells (Supplementary Figure S1A, B). PCR genotyping and sequencing confirmed the deletion of the second exon in both alleles (Supplementary Figure S1C, D). We isolated two independent cell lines (Supplementary Figure S1C, No. 2, and No.3) and analysed the proliferation of *FIGNL1* KO cells by direct cell number counting. *FIGNL1* deletion resulted in slower growth relative to that of the control (Figure 1A left). The clonogenic assay demonstrated a 3-fold decrease in colony-formation ability of *FIGNL1* KO cells relative to that in the control (29.9 ± 1.3% relative to the wild-type control) (Figure 1A middle). Assessment of viable cells by ATP-based assay showed a 2-fold reduction in the proliferation of *FIGNL1* KO cells compared with that of the control (Figure 1A right). Thus, *FIGNL1* plays a role in normal cell proliferation. We next examined the cell cycle distribution by analysing EdU incorporation, which labels ongoing DNA replication, thus cells in the S-phase, and the phosphorylation of histone H3 (Ser10) as a marker of mitosis. Although a slight increase in the percentage of S-phase cells and a slight decrease in the percentage of G1-phase cells were observed in *FIGNL1* KO cells, *FIGNL1* deletion did not affect overall cell cycle distribution (S phase: 46.8 ± 1.7% in wild-type vs 49.7 ± 0.2% in *FIGNL1* KO No. 2, G1 phase: 47.0 ± 2.2% in wild-type vs 44.0 ± 0.5% in *FIGNL1* KO No. 3) (Supplementary Figure S2A).

Based on the previous observation that FIGNL1 dissociates RAD51 from ssDNA (29), we monitored RAD51-focus formation in proliferating *FIGNL1* KO cells in the absence of exogenous DNA damage. The frequency of RAD51 focus-positive cells (with >5 foci per nucleus) drastically increased in two independent *FIGNL1* KO cells compared with that in the control (5.5 ± 2.5% in wild-type, 52.5 ± 2.7% in *FIGNL1* KO No. 2, and 39.8 ± 4.9% in *FIGNL1* KO No. 3) (Figure 1B), suggesting that FIGNL1 is required for the suppression of spontaneous RAD51 assembly in the chromatin under normal growth conditions.

A previous report showed that FIGNL1 depletion by shRNA caused reduced HR efficiency (32), implying that FIGNL1 played a positive role in HR. We examined whether impaired cell growth in *FIGNL1* KO cells reflected defective HR by analysing the sensitivity of *FIGNL1* KO cells to the Topoisomerase I inhibitor, camptothecin (CPT). CPT inhibits topoisomerase I-mediated rejoining step, which generates DSBs upon collision with a replication fork (42). The sensitivity of *FIGNL1* KO cells to CPT was comparable to that of the control (Supplementary Figure S2B). More RAD51 foci were observed in *FIGNL1* KO cells at 8 h after 1 h CPT treatment than that in control cells. Numbers of RAD51 foci in both cell lines were returned to baseline by 72 h after treatment although the baseline in *FIGNL1* KO cells were higher than that in control cells. These data suggest that *FIGNL1* is dispensable for the dissociation of RAD51 in the repair of CPT-induced DSBs (Supplementary Figure S2C). These data imply that *FIGNL1* is not essential for DSB repair under our experimental condition.

### RAD51 persists at the stalled replication fork in *FIGNL1* KO cells

**Figure 2.**
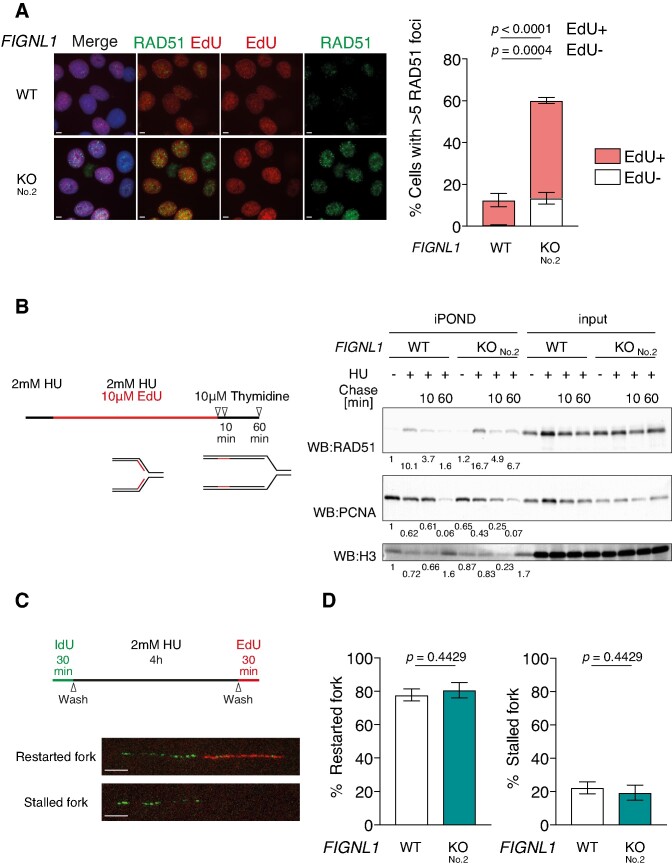
Post-replication persistence of RAD51 in *FIGNL1* KO cells. (**A**) Left, Representative images of immunofluorescence analysis of RAD51 foci (green) and EdU (red) incorporation in control U2OS cells (top) and *FIGNL1* KO cells (bottom). Right, Quantification of RAD51 focus-positive cells (>5 foci/cell) in the indicated cell lines. RAD51 focus-positive cells were classified as EdU-positive (EdU+) and EdU-negative (EdU−). More than 200 cells were counted for each sample. Data are presented as mean ± s.d. (*n* = 3, all biological independents). Scale bar = 5 μm. (**B**) Left, schematic of the iPOND assay to measure proteins associated with EdU-incorporated DNA strands under replication stress. U2OS cells were incubated in the presence of 2 mM HU and 10 μM EdU for 4 h (+HU). After 4 h of incubation, the cells were chased with 10 μM thymidine for 10 or 60 min (Chase 10 or 60). Right, Western blotting analysis of iPOND samples. Streptavidin pull-down samples and inputs were probed with the indicated antibodies. Relative band intensities to untreated wild-type samples are shown below images. (**C**) Top, schematic of DNA combing analysis to assess fork restart. U2OS cells were pulse-labelled with 20 μM IdU and incubated in the presence of 2 mM HU for 4 h. After HU treatment, the cells were washed and incubated with 100 μM EdU for 30 min. Bottom: Representative image of restarted and stalled forks. Scale bar = 5 μm. (**D**) Quantification of restarted forks (left) and stalled forks (right). More than 60 fibres were counted in each sample. Data are presented as mean ± s.d. (*n* = 3, all biological independents).

We determined the stage of the cell cycle at which FIGNL1 suppressed RAD51 assembly by assessing RAD51-focus formation with EdU incorporation. Since the proportion of EdU-positive cells was similar between the control and *FIGNL1* KO U2OS cells (Supplementary Figure S2A), most of the *FIGNL1* KO cells were not arrested in the S phase. The majority of RAD51 focus-positive cells were EdU-positive in *FIGNL1* KO cells, (*FIGNL1* KO cells; 46.7 ± 1.5% in S phase versus 13.4 ± 2.8% in non-S phase cells) (Figure 2A), suggesting that FIGNL1 is required for the dissociation of RAD51 in the S phase, thus, at the stalled replication fork. To validate this, the isolation of proteins on nascent DNA (iPOND) assay was performed under replication stress. Briefly, the cells were pulse-labelled with EdU, and the proteins associated with the replication fork were cross-linked and purified with newly synthesized EdU-labelled strands. To assess the efficiency of RAD51 assembly to stalled replication forks, the cells were treated with hydroxyurea (HU), which induces replication stress by depleting deoxyribonucleotide pools. Subsequently, to check RAD51 dissociation, the cells were washed and released into thymidine-containing media for 10 or 60 min after HU treatment (Figure 2B, left). Under unchallenged conditions, RAD51 was not detected in either the wild-type control or *FIGNL1* KO cells (−HU) (Figure 2B right). As previously reported (43), HU treatment increased RAD51 assembly at the replication fork (+HU) in both control and *FIGNL1* KO cells. Although release from replication stress by thymidine chase reduced the amount of RAD51 bound to EdU-labelled strands in the wild-type control, RAD51 persisted on newly synthesized strands in the *FIGNL1* KO cells even 1 h after release (Chase 60 min) (Figure 2B right, Supplementary Figure S3A). To detect spontaneous RAD51 persistence, cells were treated with EdU for a slightly longer time (30 min) in the absence of HU than that in Figure 2B, released in thymidine-containing media and subjected to iPOND assay. In the thymidine chase sample, *FIGNL1* KO cells presented a 2-fold increase in the amount of RAD51 at EdU-incorporated strands compared to the control (2.4 in wild-type control versus 5.3 in *FIGNL1* KO cells) (Supplementary Figure S3B). Thus, the increased spontaneous RAD51 foci in *FIGNL1* KO cells could reflect the RAD51 persistence at replication forks. We observed a similar reduction in the amount of PCNA in *FIGNL1* KO as in the control cells after the release, suggesting that replication forks seem to restart properly in both cell lines 1 h after release (Figure 2B right, Supplementary Figure S3A, B). DNA combing showed that after releasing from HU, the frequencies of restarted and stalled forks in *FIGNL1* KO cells were comparable to that of wild-type cells (restarted forks; 77.8 ± 3.6% in wild-type control vs 80.7 ± 4.6% in *FIGNL1* KO) (stalled forks; 22.2 ± 3.6% in wild-type control vs 19.3 ± 4.6% in *FIGNL1* KO) (Figure 2C, D). Similarly, EdU was incorporated in PCNA-focus positive S-phase cells to similar levels in both wild-type and *FIGNL1* KO cells (93.9 ± 3.7% in wild-type control versus 96.2 ± 1.3% in *FIGNL1* KO) (Supplementary Figure S3C). These data suggest that FIGNL1 promotes efficient dissociation of RAD51 from the chromatin after replication fork restarts and is not required for replication fork restart. The requirement of RAD51 disassembly from DNA ends for fork restart prompted us to examine the presence of post-replicative gaps. Prior to genomic DNA isolation, cells were subjected to treatment with S1 nuclease and IdU/CldU-labelled DNA fibres were analysed. The ratio of S1-treated fibre length to the untreated fibre length in wild-type control and *FIGNL1* KO cells was 0.88 and 0.61, respectively (Supplementary Figure S3D). Thus, the S1 nuclease-dependent increase in shorter fibres suggests that post-replicative gaps accumulated in *FIGNL1* KO cells.

### FIGNL1 suppresses the formation of the ultra-fine bridge

**Figure 3.**
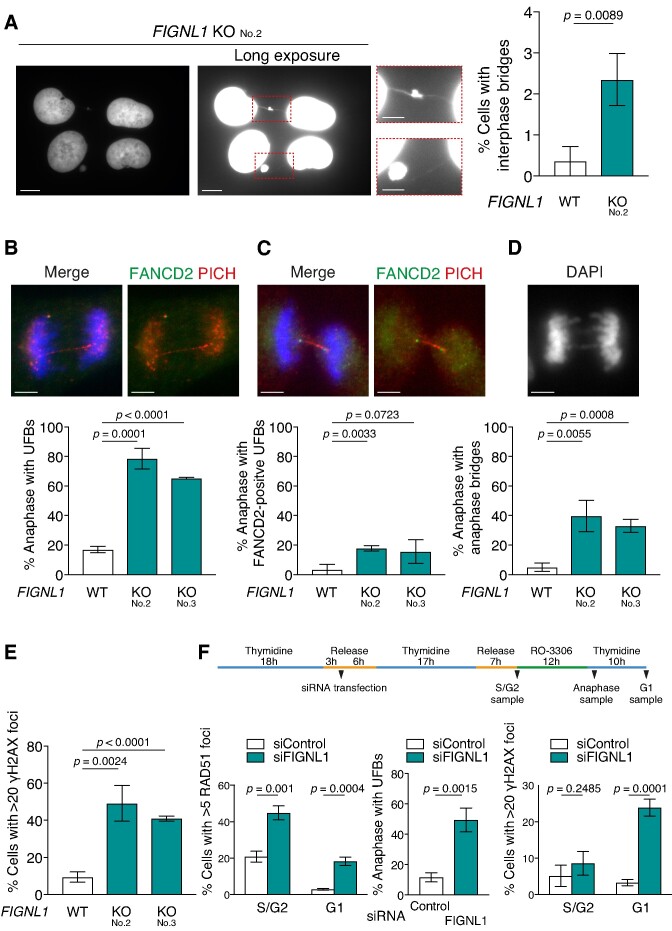
UFB formation and accumulation of DNA damage in *FIGNL1* KO cells. (**A**) Left, Representative images of chromatin bridges in *FIGNL1* KO cells. Scale bar = 10 μm. Middle: enlarged images of areas in dashed red rectangles. Scale bar = 5 μm. Right, Quantification of cells with chromatin bridges in the indicated cell lines. More than 200 cells were counted for each sample. Data are presented as mean ± s.d. (*n* = 3, all biological independents). (**B**) Top, Representative images of UFBs stained with PICH and FANCD2 antibodies. Bottom: Quantification of anaphase cells with PICH-coated UFBs in the indicated cell lines. More than 50 anaphase cells were counted for each sample. Data are presented as mean ± s.d. (*n* = 3, all biological independents). Scale bar = 5 μm. (**C**) Top, Representative images of UFBs with FANCD2 foci at the extremity. Bottom: Quantification of anaphase cells with FANCD2-positive UFBs in the indicated cell lines. More than 50 anaphase cells were counted for each sample. Data are presented as mean ± s.d. (*n* = 3, all biological independents). Scale bar = 5 μm. (**D**) Top, Representative images of anaphase bridges. Bottom, quantification of anaphase cells with anaphase bridges. More than 50 anaphase cells were counted for each sample. Data are presented as mean ± s.d. (*n* = 3, all biological independents). Scale bar = 5 μm. (**E**) Quantification of γH2AX focus-positive cells (>20 foci/cell) in the indicated cell lines. More than 200 cells were counted for each sample. Data are presented as mean ± s.d. (*n* = 3, all biological independents). (**F**) Top, Schematic representation of cell cycle synchronization and FIGNL1 depletion using siRNA. U2OS cells were synchronized using a double thymidine block. After the first round of thymidine treatment, the cells were transfected with siRNA against human FIGNL1 and control siRNA. After the second round of thymidine treatment, the cells were fixed and subjected to immunofluorescence staining at indicated points. Bottom left: Quantification of γH2AX focus-positive cells (>20 foci/cell) in the indicated cell lines. More than 200 cells were counted for each sample. Bottom middle, Quantification of anaphase cells with PICH-coated UFBs in the indicated cell lines. More than 50 anaphase cells were counted for each sample. Bottom right, Quantification of RAD51 focus-positive cells (>5 foci/cell) in the indicated cell lines. More than 200 cells were counted for each sample. Data are presented as mean ± s.d. (*n* = 3, all biological independents).

The above results suggest that inappropriate RAD51 persistence on chromatin may affect cellular events after DNA replication. Notably, DNA staining with DAPI showed increased formation of interphase nuclear bridges between adjacent *FIGNL1* KO cells (0.36 ± 0.36% in wild-type control versus 2.4 ± 0.6% in *FIGNL1* KO, *P* < 0.01) (Figure 3A). As the increased formation of interphase nuclear bridges implicated improper chromosome segregation during mitosis, we investigated chromosome connections during anaphase. Consistent with the increase in nuclear bridge formation, we observed an increased formation of the ultra-fine bridge (UFB), which is marked by PICH localization on the bridge, in *FIGNL1* KO cells compared to that in wild-type control (17.0 ± 2.1% in wild-type control, 78.6 ± 7.0% in *FIGNL1* KO No.2, 65.3 ± 0.4% in *FIGNL1* KO No. 3) (Figure 3B, Supplementary Figure S4A). The majority of PICH-positive UFBs in *FIGNL1* KO cells were negative for FANCD2, which indicates incomplete replication (Figure 3B, C). Furthermore, the frequency of cells with a typical anaphase bridge detected as DAPI thread was also significantly increased in *FIGNL1* KO cells (5.0 ± 2.8% in wild-type control, 39.7 ± 10.6% in *FIGNL1 KO* No. 2, *P* < 0.001, 33.0 ± 4.5% in *FIGNL1* KO No. 3, *P* < 0.001) (Figure 3D). These data indicate that FIGNL1 is critical to suppress the formation of anaphase bridges and UFB, suggesting that RAD51 persistence leads to the formation of these chromosome bridges. Moreover, the frequency of anaphase cells with UFBs was significantly higher than that of cells with typical anaphase bridges, implying that the loss of *FIGNL1* primarily causes UFB formation.

UFB formation leads to chromosome breakage during the next round of the cell cycle (10,44). To examine if *FIGNL1* KO induces chromosome breaks, we monitored the formation of γH2AX foci as a marker of DSB and micronuclei (MN), which could be derived from chromosome fragmentation. The *FIGNL1* KO cells showed a significantly increased frequency of spontaneous γH2AX-positive cells under normal conditions (γH2AX; 9.4 ± 2.8% in wild-type control, 49.1 ± 9.6% in *FIGNL1* KO No. 2, *P* < 0.005, 40.9 ± 1.4% in *FIGNL1* KO No. 3, *P* < 0.0001) (Figure 3E). Moreover, the frequency of MN-positive cells was significantly higher by approximately 8.5-fold in the *FIGNL1* KO cells than in the wild-type control (2.7 ± 0.6% in wild-type control versus 22.9 ± 2.7% in *FIGNL1 KO*, *P* < 0.001) (Supplementary Figure S5D). These data imply that FIGNL1 suppresses chromosome fragmentation by preventing UFB formation. Since U2OS cells maintain telomeres by homologous recombination-mediated pathways known as alternative lengthening of telomeres (ALT), we examined whether the deletion of *FIGNL1* induces UFB formation specifically in ALT cells. To this end, we assessed the formation of RAD51-focus, UFB and γH2AX-focus in non-ALT HeLa cells. Similar to U2OS cells, we observed that the deletion of *FIGNL1* increased UFBs, RAD51 focus-positive cells, and γH2AX-positive cells in the HeLa cell background (Supplementary Figure S4B–F). These data support that the deletion of FIGNL1 induces UFB formation in an ALT-independent manner.

Next, we examined whether DSBs found in the absence of *FIGNL1* are produced by mitotic progression by monitoring γH2AX- and RAD51-focus formation during S/G2 and the subsequent G1 phase of the cell cycle and UFB formation in anaphase following FIGNL1 depletion using siRNA (Supplementary Figure S5A). The cells were synchronized using a double thymidine block. After the first round of thymidine treatment, the cells were transfected with siRNA against human FIGNL1 to deplete FIGNL1 in the next S phase. After the second round of thymidine treatment, the cells were incubated to progress into the S and G2 phases and fixed. For the G1 phase sample, the cells after the second round of thymidine treatment were synchronized in the G2 phase and released into thymidine-containing media to progress into the G1 phase and fixed (Figure 3F top). In S/G2 phase-synchronized cells, the frequency of RAD51 focus-positive cells was significantly increased in FIGNL1-depleted cells compared with that in control cells (44.8 ± 3.8% in FIGNL1-depleted cells versus 20.9 ± 3.0% in control siRNA-transfected cells, *P* = 0.001) (Figure 3F, bottom left), suggesting that FIGNL1 depletion induces RAD51 accumulation on chromatin in the S-phase (Figure 2A). However, the frequency of γH2AX-positive cells in S/G2 phase FIGNL1-depleted cells was indistinguishable from that in control cells, implying that the increased RAD51-focus formation in FIGNL1-depleted cells is not caused by the accumulation of spontaneous DSBs or unrepaired DSBs (8.6 ± 3.3% in FIGNL1-depleted cells versus 5.1 ± 2.9% in control siRNA-transfected cells, *P* = 0.2485) (Figure 3F, bottom right). Consistent with UFB formation in *FIGNL1* KO cells, UFB formation was induced by FIGNL1 depletion (Figure 3F, bottom middle). The frequency of γH2AX-positive cells in the FIGNL1*-*depleted cells was significantly increased in the next G1 phase (23.9 ± 2.3% in FIGNL*1*-depleted cells versus 3.3 ± 0.9% in control siRNA-transfected cells, *P* < 0.001) (Figure 3F, right). These data suggest that mitotic progression is required to induce DSBs in FIGNL1-depleted cells.

Since the deletion of *FIGNL1* slightly increased the sensitivity to transient HU treatment (Supplementary Figure S5B), we next assessed UFB and MN formation after release from transient replication stress. To assess the effect of replication stress on UFB formation, the cells were synchronized using a double thymidine block and treated with HU for 4 h right after the second round of thymidine treatment. After release from HU treatment, the cells were fixed in anaphase. Transient HU treatment increased UFB formation in the control and *FIGNL1* KO cells (Supplementary Figure S5C). For MN analysis, the cells were treated with HU for 4 h and fixed 24 and 48 h after HU release. Transient HU treatment induced MN at 24 and 48 h after release in control and *FIGNL1* KO cells (Supplementary Figure S5D). As the replication fork restarts within 1 h after HU treatment (Figure 2B–D), these observations suggest that transient fork stalling and subsequent restart potentiate the formation of UFB and MN.

### Accumulation of RAD51 at the telomere and centromere leads to UFB formation

**Figure 4.**
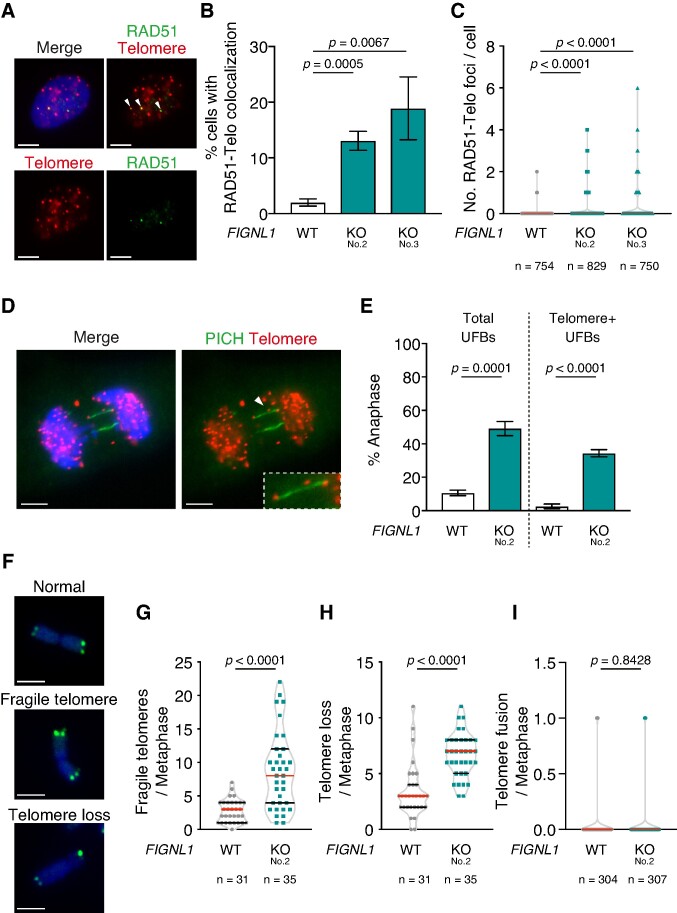
FIGNL1 suppresses telomere-mediated UFB formation and telomere instability. (**A**) Representative images of immunofluorescence-FISH analysis of RAD51 and telomere foci in *FIGNL1* KO cells. The arrowheads indicate colocalization of RAD51 and telomere signals. Scale bar = 5 μm. (**B**) Quantification of cells with RAD51 foci colocalized with telomere signal in the indicated cell lines (shown as RAD51–Telo colocalization). More than 200 cells were counted for each sample. Data are presented as mean ± s.d. (*n* = 3, all biological independents). (**C**) Quantification of RAD51 foci colocalized with telomere signals. Data are presented as median (red line) with IQR (black dashed line). (**D**) Representative images of immunofluorescence-FISH analysis of PICH and telomere in *FIGNL1* KO cells. Scale bar = 5 μm. The inset shows a magnified image of the UFB. (**E**) Quantification of anaphase cells with telomere signals on UFBs in the indicated cell lines. More than 50 cells were counted for each sample. Data are presented as mean ± s.d. (*n* = 3, all biological independents). (**F**) Representative image of a metaphase chromosome with fragile telomeres and telomere loss. Scale bar = 5 μm. (**G–I**) Quantification of fragile telomeres (G), telomere loss (H), and telomere fusion (I) per metaphase in the indicated cell lines. Data are presented as median (red line) with IQR (black dashed line).

RAD51 persistence under normal DNA replication in *FIGNL1* KO cells could imply RAD51 accumulation at chromosomal loci with intrinsic replication difficulties. To verify this hypothesis, we monitored RAD51-focus formation and UFB formation at telomeres with repetitive DNA sequences, which are a particular challenge to genome stability due to the propensity to form DNA secondary structures that hinder replication progression (45,46). The *FIGNL1* KO cells showed increased frequencies of cells with colocalization of RAD51 foci and telomere FISH signals (Figure 4A, B; 2.0 ± 0.7% in wild-type control, 13.1 ± 1.7% in *FIGNL1* KO No. 2, 18.9 ± 5.6% in *FIGNL1* KO-No. 3). The number of RAD51 foci colocalized with telomeres also increased in the KO cells (Figure 4C, Supplementary Figure S6A) (Figure 4C; 0.02 ± 0.15 in wild-type control, 0.18 ± 0.53 in *FIGNL1* KO No.2, 0.32 ± 0.78 in *FIGNL1* KO No.3). Double-staining of PICH and telomeres revealed that the majority of the *FIGNL1* KO cells harboured telomere signals on the UFB (2.6 ± 1.4% in wild-type control versus 34.3 ± 2.1% in *FIGNL1* KO) (Figure 4D, E) and 44.7% of UFBs had telomere signal (No. of telomere-positive UFB/anaphase 0.34 ± 0.02 and No. of telomere-negative UFB / anaphase 0.42 ± 0.08) (Supplementary Figure S6C), suggesting that nearly half of the linkage between sister chromatids occurs at telomeres. If the linkage between telomeres is associated with UFB formation, the instability of the telomere sequence could be increased by *FIGNL1* deletion. To test this hypothesis, we examined telomere instability in *FIGNL1* KO cells using telomere FISH on metaphase spreads. *FIGNL1* KO led to significantly increased telomere fragility or loss, which were observed as multiple FISH signals or loss of FISH signals at chromosome ends, respectively (fragile telomere; 2.77 ± 1.69 in wild-type control vs 8.69 ± 5.73 in *FIGNL1* KO, *P* < 0.0001) (telomere loss; 3.42 ± 2.43 in wild-type control vs 6.66 ± 1.97 in *FIGNL1* KO, *P* < 0.0001) (Figure 4F, G, H). Increased telomere instability in *FIGNL1* KO cells shows the role of FIGNL1 in protecting telomere integrity under normal conditions. Moreover, we observed connected sister telomere FISH signals (inter-sister bridge-like signals) and symmetric elongated telomere FISH signals (thread-like telomere signals) between sister chromatids were increased in *FIGNL1* KO cells (Supplementary Figure S6D, E). These data suggest a linkage between telomeres in the absence of *FIGNL1* could induce telomere instability. In contrast, changes in the frequency of telomere fusion were not observed in the KO cells (Figure 4I), implying telomere fusion-independent UFB formation in *FIGNL1* KO cells.

**Figure 5.**
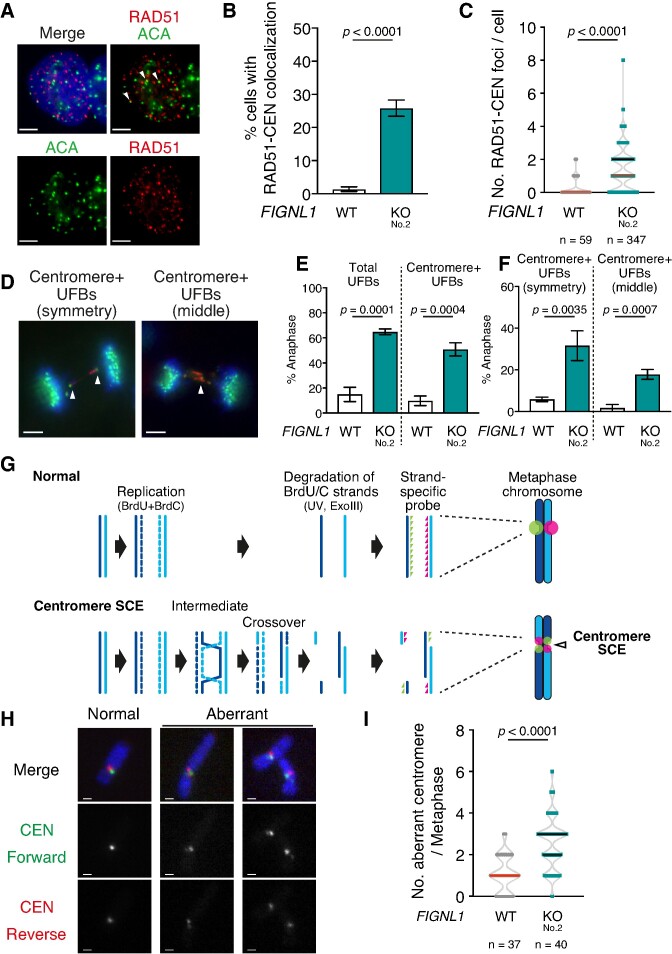
FIGNL1 suppresses centromere mediated UFB formation and centromere SCE. (**A**) Representative images of immunofluorescence analysis of RAD51 and centromere foci in *FIGNL1* KO cells. Arrowheads indicate colocalization of RAD51 and centromere signals. Scale bar = 5 μm. (**B**) Quantification of cells with RAD51 foci colocalized with centromere signals in the indicated cell lines. More than 200 cells were counted for each sample. Data are presented as mean ± s.d. (*n* = 3, all biological independents). (**C**) Quantification of RAD51 foci colocalized with centromere signals. Data are presented as median (red line) with IQR (black dashed line). (**D**) Representative images of immunofluorescence analysis of PICH (red) and centromere (green) in *FIGNL1* KO cells. Scale bar = 5 μm. (**E**) Quantification of anaphase cells with UFBs and anaphase cells with centromere signals on PICH-stained UFBs in the indicated cell lines. More than 50 cells were counted for each sample. Data are presented as mean ± s.d. (*n* = 3, all biological independents). (**F**) Quantification of anaphase cells with twin centromere signals at the end of UFBs and centromere signals in the middle region of UFBs in indicated cell lines. More than 50 cells were counted for each sample. Data are presented as mean ± s.d. (*n* = 3, all biological independents). (**G**) Schematic of centromere CO-FISH to assess sister chromatid exchanges (SCEs). After the incorporation of BrdU and BrdC, metaphase spreads were prepared. BrdU/C incorporated DNA strands were degraded by UV and Exo III treatment. Undegraded DNA strands were hybridized with CENPB-box specific forward (green arrow heads) and reverse (red arrow heads) FISH probes. Sister centromeres on normal chromosomes can be distinguished as individual green and red signals while centromeres with SCEs have green and/or red signals on both sister chromatids. (**H**) Left, representative image of a normal CO-FISH pattern. Middle and right, Representative image of chromosome with SCEs (Aberrant). Scale bar = 1 μm. (**I**) Quantification of aberrant centromeres per metaphase in the indicated cell lines. Chromosomes with red and/or green signals on both sister chromatids were counted. Data are presented as median (red line) with IQR (black dashed line).

We also examined UFB formation at the centromere, which is another chromosome locus with repetitive DNA sequences and an unstable nature (47), Similar to the telomeres, increased localization of RAD51 at centromeres was observed (Figure 5A-C, Supplementary Figure S6B) as an increase in the frequency of cells with colocalization of RAD51 and centromere (ACA) (Figure 5B; 1.4 ± 0.7% in wild-type control versus 25.9 ± 2.5% in *FIGNL1 KO*, *P* < 0.0001) and increased number of RAD51 foci colocalized with centromere signals in *FIGNL1 KO* cells (Figure 5C; 1.25 ± 0.45 in wild-type control versus 1.96 ± 1.01 in *FIGNL1* KO, *P* < 0.05). The presence of centromere signals on UFBs was observed in *FIGNL1* KO cells (9.7 ± 3.8% in wild-type control versus 50.8 ± 5.3% in *FIGNL1* KO, *P* < 0.001) (Figure 5D, E). We observed two types of centromere signals on UFBs: symmetrical centromere signals at the ends of the bridge (Figure 5D, left) and centromere signals in the middle region of the bridge (Figure 5D, right). UFBs with symmetrical centromere signals reflect a linkage between the arm regions of sister chromatids, suggesting that the linkage in the UFB occurs between the same region in each arm of the sister chromatids. UFBs with centromere signals in the middle region reflect the linkage between the centromeres. Both types of UFBs were significantly increased in *FIGNL1* KO cells, suggesting that a linkage between centromeres or arm regions induces UFB formation in *FIGNL1* KO cells (symmetry; 5.8 ± 1.1% in wild-type control versus 31.6 ± 7.2% in *FIGNL1* KO, *P* < 0.005) (middle; 1.6 ± 1.7% in wild-type control versus 17.8 ± 2.4% in *FIGNL1* KO, *P* < 0.001) (Figure 5F, Supplementary Figure S6F). Furthermore, *FIGNL1* deletion induced centromere instability including chromosome breaks and gaps (Supplementary Figure S6G). Thus, replication fork stalling at chromosomal loci with replication difficulties such as telomeres and centromeres may often trigger the persistence of RAD51 in the absence of *FIGNL1* and generate an unresolved linkage between sister chromatids.

Next, we investigated whether persistent RAD51 in *FIGNL1* KO cells induces strand exchange between repetitive sequences by assessing the sister-chromatid exchange (SCE) at the centromere using chromosome-orientation fluorescence *in situ* hybridization (CO-FISH) (41) (Figure 5G). After treating the cells with BrdU during a single round of the cell cycle, BrdU-incorporated DNA strands were degraded and centromeric repeats were hybridized with strand-specific centromeric probes. Sister centromeres in normal chromatids were observed as side-by-side green and red signals (Figure 5G, H). In contrast to that in wild-type control, both sister chromatids in the *FIGNL1* KO cells had centromeric signals of the same colour (aberrant centromere), which indicates centromeric SCE (41,48) (1.08 ± 0.86 in wild-type control versus 2.6 ± 1.30 in *FIGNL1* KO, *P* < 0.001) (Figure 5H, I). Thus, FIGNL1 suppresses centromeric SCEs, implying that persistent RAD51 in *FIGNL1* KO cells promotes the formation of recombination intermediate-like structures between sister chromatids.

### FIGNL1 and resolvase are epistatic in terms of suppressing UFB formation

UFBs are classified into five groups: FS-UFBs, T-UFBs, C-UFBs, R-UFBs, and HR-UFB. T-UFBs, C-UFBs, and R-UFBs are generated by telomere fusion and DNA catenanes of centromeric and ribosomal regions, respectively, and hence are specific to genomic loci. FS-UFBs generated by incomplete replicative regions have FANCD2 foci at the bridge termini. UFBs observed in *FIGNL1* KO cells were neither specific to telomeres nor centromeres (Figures 4D, E, 5D–F) and were often FANCD2-negative (Figure 3B, C). Hence, we tested whether the UFBs detected in *FIGNL1* KO cells are HR-UFB, which are usually observed in Holliday-junction resolvase-deficient cells (10), by analysing UFB formation after depleting both GEN1 and MUS81 resolvases in *FIGNL1* KO cells. Consistent with the previous observation (10), co-depletion of GEN1 and MUS81 led to UFB formation (25.2 ± 5.0% in control cells versus 52.5 ± 6.7% in GEN1- and MUS81-depleted cells) (Supplementary Figure S7A). The frequency of anaphase cells with UFBs in resolvase-depleted *FIGNL1* KO cells did not significantly differ from that in *FIGNL1* KO cells (57.5 ± 3.2% in *FIGNL1* KO cells versus 67.0 ± 6.5% in GEN1- and MUS81-depleted *FIGNLl1* KO cells, *P* = 0.0557) (Supplementary Figure S7A). These data indicate that FIGNL1 and resolvases are epistatic in terms of suppressing UFB formation, suggesting that the loss of *FIGNL1* may induce the formation of HR-UFB-like bridges. Indeed, the exogenous expression of GEN1 significantly reduced UFBs in *FIGNL1* KO cells (61.0 ± 2.3% in *FIGNL1* KO cells versus 25.1 ± 6.0% in FLAG-GEN1-expressing *FIGNL1* KO cells) (Supplementary Figure S7B).

### Inhibition of RAD51 activity rescues defects in *FIGNL1* KO cells

**Figure 6.**
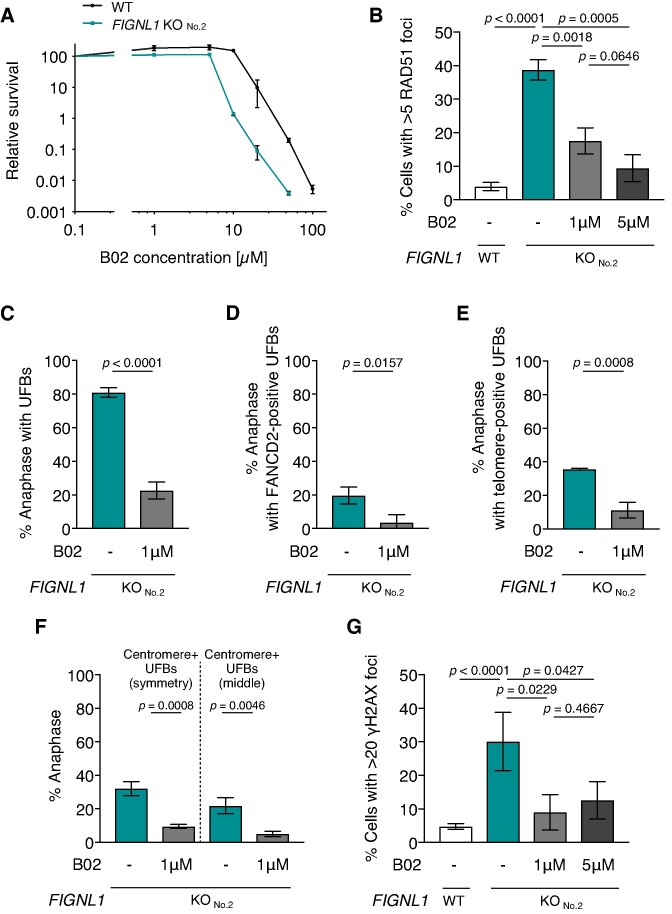
Inhibition of RAD51 rescues defects in *FIGNL1* KO cells. (**A**) Relative survival of control U2OS cells and *FIGNL1* KO cells exposed to the indicated concentrations of the RAD51 inhibitor, B02. The cells were incubated in the presence of the indicated concentration of B02 for 24 h. After 7 days, the cells were subjected to a cell titer Glo assay, and relative luminescence as an indicator of the number of viable cells is shown. Data are presented as mean ± s.d. (n = 3, all biological independents). (**B**) Quantification of RAD51 focus-positive cells (> 5 foci/cell) in control U2OS cells and *FIGNL1* KO cells treated with the indicated concentration of B02. More than 200 cells were counted for each sample. Data are presented as mean ± s.d. (*n* = 3, all biological independents). (**C**) Quantification of anaphase cells with UFBs in *FIGNL1* KO cells treated with 1 μM B02. More than 50 anaphase cells were counted for each sample. Data are presented as mean ± s.d. (*n* = 3, all biological independents). (**D**) Quantification of anaphase cells with FANCD2-positive UFBs in *FIGNL1* KO cells treated with 1 μM B02. More than 50 anaphase cells were counted for each sample. Data are presented as mean ± s.d. (*n* = 3, all biological independents). (**E**) Quantification of anaphase cells with telomere signals on UFBs in the indicated cell lines. More than 50 cells were counted for each sample. Data are presented as mean ± s.d. (*n* = 3, all biological independents). (**F**) Quantification of anaphase cells with twin centromere signals at the end of UFBs and centromere signals in the middle region of UFBs in indicated cell lines. More than 50 cells were counted for each sample. Data are presented as mean ± s.d. (*n* = 3, all biological independents). (**G**) Quantification of γH2AX focus-positive cells (>20 foci/cell) in control U2OS cells and *FIGNL1* KO cells treated with the indicated concentrations of B02. More than 200 cells were counted for each sample. Data are presented as mean ± s.d. (*n* = 3, all biological independents).

We hypothesized that the inappropriate persistence of RAD51 induces UFB formation by promoting the strand exchange between sister chromatids. To test this hypothesis, we examined whether inhibition of RAD51 activity rescued the defects in *FIGNL1* KO cells by assessing RAD51- and γH2AX-focus formation and UFB formation in the presence of B02, a RAD51 inhibitor. B02 inhibits the DNA-binding activity of RAD51 both *in vivo* and *in vitro* (49,50). Treatment with a high concentration of B02 completely inhibits RAD51 and leads to genome instability because of defective HR and replication fork protection (49,50). Hence, we first determined the optimal concentration of B02 for the rescue experiments. Significant growth defects were observed at 20, 50 and 100 μM B02, whereas 1, 5 and 10 μM B02 did not affect the proliferation of control U2OS cells (Figure 6A). *FIGNL1* KO cells exhibited increased sensitivity to 10, 20 and 50 μM B02, implying an additional role of RAD51 in the absence of FIGNL1. For the rescue experiments, we decided to use 1 and 5 μM B02 for subsequent experiments. The treatment with 1 and 5 μM of B02 significantly reduced spontaneous RAD51-focus formation, which is a characteristic of *FIGNL1* KO cells (untreated *FIGNLl1* KO 38.7 ± 3.1%, 1 μM 17.6 ± 3.9%, *P* < 0.005, 5 μM 9.5 ± 4.0%, *P* < 0.001) (Figure 6B, Supplementary Figure S9C). The treatment with 1 μM B02 significantly decreased UFB formation with and without FANCD2 signals in *FIGNL1* KO cells (untreated 80.9 ± 2.9% versus 1 μM 22.6 ± 5.0%, *P* < 0.001) (Figure 6C, D, Supplementary Figure S8A). Moreover, both UFBs with telomere signals and centromere signals were reduced by 1 μM B02 treatment (Figure 6E, F, Supplementary Figure S8B, C). Thus, UFBs in *FIGNL1* KO cells are suppressed by reducing RAD51 activity. Both FANCD2-positive and -negative UFBs are generated in a RAD51-dependent manner. Furthermore, B02 treatment significantly decreased the frequency of γH2AX-positive cells (untreated *FIGNL1 KO* 30.1 ± 8.7%, *P* < 0.05, 1 μM 9.0 ± 5.3%, 5 μM 12.6 ± 5.6%, *P* < 0.05) (Figure 6G). These results support our hypothesis that the inappropriate persistence of RAD51 filaments caused by the loss of *FIGNL1* induces UFB formation.

### FIGNL1 suppresses UFB formation by dissociating inappropriate RAD51 filaments through its FxxA motif

**Figure 7.**
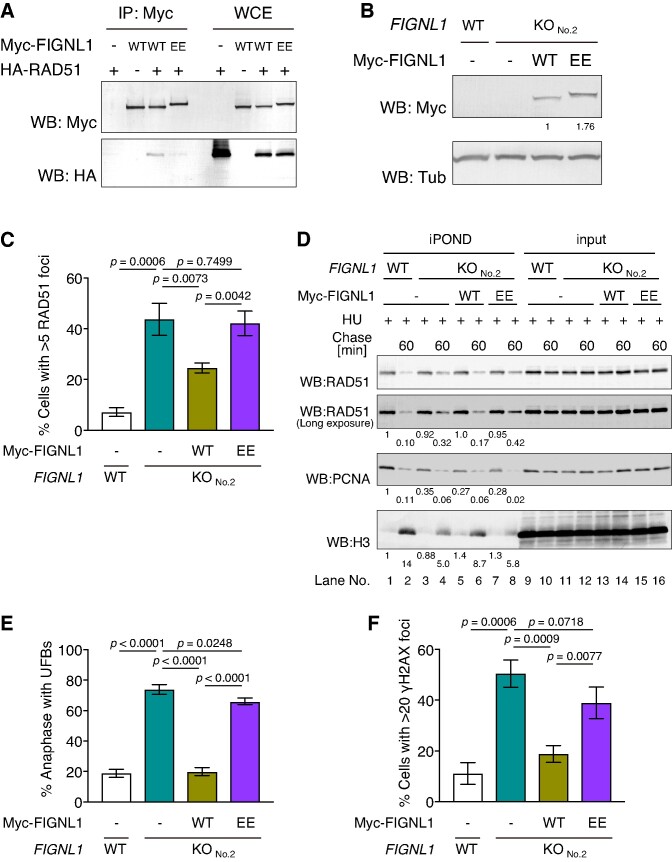
FIGNL1-RAD51 interaction is required to suppress RAD51 accumulation and UFB formation. (**A**) Western blotting of Myc-FIGNL1 immunoprecipitates. HA-RAD51 and Myc-FIGNL1 variants (wild-type or EE mutant) were co-expressed in 293T cells and immunoprecipitated using an anti-Myc antibody. The immunoprecipitates (IP) and whole cell extract (WCE) were stained with the indicated antibodies. (**B**) Western blotting analysis of Myc-FIGNL1 variant-expressing cell lines. Myc-FIGNL1 or Myc-FIGNL1-EE were expressed in the indicated cell lines. The WCEs were probed with the indicated antibodies. Relative band intensities to Myc-FIGNL1 are shown below images. (**C**) Quantification of RAD51 focus-positive cells (>5 foci/cell) in control U2OS cells and *FIGNL1* KO cells with or without expression of Myc-FIGNL1 mutants. More than 200 cells were counted for each sample. Data are presented as mean ± s.d. (*n* = 3, all biological independents). (**D**) Western blotting analysis of the iPOND samples. Cells were incubated in the presence of 2 mM HU and 10 μM EdU for 4 h (+HU). After 4 h of incubation, the cells were chased with 10 μM thymidine for 60 min (Chase 60). iPOND samples and inputs were probed with the indicated antibodies. Relative band intensities to non-treated wild-type samples are shown below images. (**E**) Quantification of anaphase cells with UFBs in control U2OS cells and *FIGNL1* KO cells with or without the expression of Myc-FIGNL1 mutants. More than 50 anaphase cells were counted for each sample. Data are presented as mean ± s.d. (*n* = 3, all biological independents). (**F**) Quantification of γH2AX focus-positive cells (>20 foci/cell) in control U2OS cells and *FIGNL1* KO cells with or without Myc-FIGNL1 mutant expression. More than 200 cells were counted for each sample. Data are presented as mean ± s.d. (*n* = 3, all biological independents).

Next, we examined whether FIGNL1 directly suppressed UFB formation and DNA damage by dissociating inappropriate RAD51 filaments. Our previous observation showed that purified FIGNL1 interacts with RAD51 through its conserved FxxA motif, and the substitution of conserved phenylalanine and alanine with glutamate (EE mutation; F295E, A298E) in the FxxA motif reduces the interaction with RAD51 and its RAD51 disassembly activity (29). To confirm whether the FxxA motif is required for the interaction with RAD51 in the cell, we performed a co-immunoprecipitation assay using cell lysates from Myc-FIGNL1- and HA-RAD51-expressing cells. Consistent with our previous observation (29), the *FIGNL1-EE* mutation greatly reduced the interaction with RAD51 (Figure 7A). We expressed Myc-FIGNL1 or Myc-FIGNL1-EE in *FIGNL1* KO cells and assessed RAD51- and γH2AX-focus formations and UFBs (Figure 7B). The expression of Myc-FIGNL1 but not of Myc-FIGNL1-EE significantly reduced the frequency of RAD51 focus-positive cells, suggesting that FIGNL1 dissociates inappropriate RAD51 filaments through its interaction with RAD51 (24.6 ± 1.9% in Myc-FIGNL1-expressing *FIGNL1* KO cells, 42.2 ± 4.8% in Myc-FIGNL1-EE-expressing *FIGNL1* KO cells, *P* < 0.005) (Figure 7C, Supplementary Figure S9D). Additionally, iPOND analysis confirmed that at 1 h after release from HU treatment, the RAD51 level detected in Myc-FIGNL1-EE-expressing cells was higher than that in Myc-FIGNL1-expressing cells (relative band intensities to untreated wild-type control: 0.17 in Myc-FIGNL1-expressing cells versus 0.42 in Myc-FIGNL1-EE-expressing cells) (Figure 7D, lanes 6, 8). Myc-FIGNL1 expression reduced the frequency of γH2AX-positive cells and UFB formation, whereas Myc-FIGNL1-EE expression did not suppress these defects in *FIGNL1* KO cells (UFB; 20.0 ± 2.7% in Myc-FIGNL1-expressing *FIGNL1* KO cells, 66.1 ± 2.2% in Myc-FIGNL1-EE-expressing *FIGNL1* KO cells, *P* < 0.001) (γH2AX; 18.8 ± 3.3% in Myc-FIGNL1-expressing *FIGNL1* KO cells, 39.0 ± 6.2% in Myc-FIGNL1-EE-expressing *FIGNL1* KO cells, *P* < 0.01) (Figure 7E, F). Thus, FIGNL1 suppresses UFB formation by dissociating RAD51 from chromatin.

## Discussion

In this study, we identified a novel type of genome instability induced by persistent RAD51 assembly. Inappropriate RAD51 persistence caused by deletion of the AAA+ ATPase *FIGNL1* led to RAD51-mediated UFB formation, which induced DNA damage and growth defects. In the absence of *FIGNL1*, RAD51 recruited to the stalled replication forks persisted after the restart of replication forks and promoted the strand exchange between sister chromatids inappropriately. Thus, FIGNL1 is a potential regulator of functions of RAD51 in HR and DNA replication. Our findings provide new insight into the relationship between the regulation of RAD51 assembly/disassembly and the maintenance of genome stability.

In this study, human *FIGNL1*-deficient cells showed RAD51 persistence at post-replicated regions and defective chromosome segregation. Notably, increased UFBs and DNA damage in *FIGNL1*-deficient cells were suppressed by the inhibition of RAD51 filament formation (Figure 6), suggesting that RAD51 filament persistence *per se* induces chromosome segregation error. FIGNL1 depletion induced RAD51-focus formation but not DSB formation in the S/G2 phase, and rather DSBs were generated after mitosis (Figure 3F). These DSBs can potentially induce genome instability including typical anaphase bridges through chromosome fusions and FS-UFBs by preventing replication fork progression (Figure 3C, D, Supplementary Figure S5D). Thus, the persistence of RAD51 leads to extensive genome instability, similar to the BFB cycle. A recent study reported that deleting mouse *FIGNL1* resulted in an embryonic lethal phenotype (51). Our findings that the deletion of human *FIGNL1* results in the accumulation of UFBs and extensive genome instability can potentially explain the mouse phenotypes, in part, and the toxicity of RAD51 overexpression associated with some types of cancer (24).

Previous studies have suggested five types of UFBs caused by specialized DNA structures or specified loci: FS-UFBs, C-UFBs, R-UFBs, T-UFBs and HR-UFBs (4). The majority of UFBs in *FIGNL1* KO cells were FANCD2-negative, suggesting that the UFBs observed in the KO cells do not result from incomplete replication (Figure 3C). We found an increased incidence of both telomere- and centromere-mediated UFBs in *FIGNL1* KO cells (Figures 3 and 4). As T-UFBs, C-UFBs and R-UFBs are locus-specific UFBs and result from different mechanisms, UFBs in *FIGNL1* KO cells are distinct from previously reported T-UFBs and C-UFBs. Indeed, *FIGNL1* KO cells did not present telomere fusion (Figure 4I). Thus, UFBs in *FIGNL1* KO cells can be induced by the common property of chromosomal loci with intrinsic replication difficulties, such as transient stalling of replication forks. HR-UFBs are caused by unresolved recombination intermediates, which are produced from DSBs in resolvase (MUS81 and GEN1)-deficient cells (10). Similar levels of UFBs were observed in *FIGNL1*-deficient, resolvase-deficient, and *FIGNL1*, *MUS81*, and *GEN1* triple-deficient cells (Supplementary Figure S7A), suggesting that FIGNL1 and resolvases suppress UFB formation in the same pathway. Furthermore, the reduction of UFBs by the overexpression of GEN1 in *FIGNL1* KO cells may reflect the formation of recombination intermediate-like structures in *FIGNL1* KO cells. These data imply that FIGNL1 suppresses UFB formation by preventing RAD51-mediated sister chromatid entanglements. To test this possibility, we examined the localization of RAD51 on UFBs. We stained UFBs with the RAD51 antibody. However, no RAD51 foci were observed on mitotic chromosomes. Previous studies failed to detect RAD51-focus formation in anaphase cells (41,52). Instead, we observed FANCD2 foci in the middle region of UFBs in *FIGNL1* KO cells, which was suppressed by B02 treatment or Myc-FIGNL1 expression (Supplementary Figure S8D-G). Given that FANCD2 interacts with SLX4 (53), our observation suggests the existence of an unresolved recombination intermediate-like structure in the middle region of UFBs. RAD51 may partially disassemble from the recombination intermediate after strand invasion by the action of other RAD51-dismantling enzymes or an undetectable amount of RAD51 is sufficient to cause strand invasion in *FIGNL1* KO cells. Increased RAD51 foci in the S/G2 phase were significantly reduced in the next G1 phase, even in *FIGNL1* KO cells.

The phenotypes of *FIGNL1* KO cells under physiological conditions included: (i) *FIGNL1* deletion leads to UFB formation, (ii) RAD51 persistence post-replicated region, (iii) accumulation of gaps on nascent DNA strands and (iv) UFB formation was suppressed by RAD51 inhibition. Given that fork restart was not affected by *FIGNL1* deletion (Figure 2B–D), the cells could restart forks even in the presence of RAD51-coated DNA. Fork repriming is one possible method of restarting the fork. RAD51-coated DNA strands could be recognized as an obstacle and replication restarts downstream of persistent RAD51 by repriming, which could be mediated by PrimPol (54). RAD51 filament adjacent to a gap may initiate homology search and strand invasion, analogously to HR (Supplementary Figure S10). After strand invasion, persistent RAD51 could inhibit DNA synthesis from the invaded DNA strand and thus prevent resolution of the intermediate-like structure. This unresolved entanglement can serve as a physical linkage between sister chromatids and causes UFB formation. Another possibility is the existence of backup enzyme(s) for RAD51 dismantling. In the absence of repriming, RAD51 polymers on the DNA end may be partially removed by other RAD51-dismantling enzymes or DNA transacting enzymes as a backup pathway (Supplementary Figure S10). This removal allows fork restart. The residual RAD51 induces the formation of recombination intermediated-like structures and UFBs. In the next cell cycle, the remaining small amount of RAD51 on the template strand may prevent DNA replication and generate replicative gaps.

Our finding that FIGNL1 removes RAD51 after replication fork restart is intriguing, considering previous observations. We previously reported that FIGNL1 depletion rescues a defect in RAD51 assembly to CPT-induced DSBs in the absence of SWSAP1, a RAD51 paralogue (29). At DSBs, SWSAP1 stabilizes the RAD51 filament by inhibiting FIGNL1’s RAD51 dismantling activity through the physical interaction. In DNA replication, the loss of the SWSAP1–SWS1 complex causes defects in fork restart but not in RAD51-mediated protection of the nascent strand (55). Previous studies suggested that SWSAP1 functions in the late step of fork restart, such as strand invasion of RAD51 filament into the template strands. Since FIGNL1 dissociates RAD51 from the post-replicated region, the FIGNL1 and SWSAP1-SWS1 complex may separately function in the process of fork restart. These different functions at DSBs and replication forks might be achieved by timely recruitment through protein-protein interaction. Further studies on the mechanisms of the recruitment and the interaction will be required to determine distinct processes between DSB repair and fork restart. BLM, FBH1, RECQL5, PARI, RADX can dissociate RAD51 from DNA (27,28,30,31,56). BLM interacts with RPA at replication forks and is necessary for proper fork restart (27,57). FBH1 is recruited to the replication fork through interaction with PCNA or ssDNA and has a role in the regulation between translesion synthesis (TLS) and HR (31,58). PARI interacts with PCNA and processes stalled replication forks (30,59). RADX removes excessive RAD51 from replication forks and regulates fork reversal by dissociating RAD51 (56). In contrast to these RAD51-dismantling enzymes that are recruited to stalled replication forks to promote fork restart, FIGNL1 is not required for fork restart but is critical for the dissociation of RAD51 from the post-replicated region. Thus, we propose that FIGNL1 is a specialized RAD51-dismantling enzyme, which functions after fork restart to ensure proper chromosome segregation.

## Supplementary Material

gkae263_Supplemental_Files

## Data Availability

The data underlying this article are available in the article and in its online supplementary material. Further data underlying this article will be shared on reasonable request to the corresponding author.
