# Supplementary material for: Human AAA+ ATPase FIGNL1 suppresses RAD51-mediated ultra-fine bridge formation
Source: Nucleic Acids Res. 2024 Apr 10;52(10):5774–91. doi: 10.1093/nar/gkae263 (PMC11162793; doi:10.1093/nar/gkae263)
Supplement: gkae263_Supplemental_Files [file gkae263_supplemental_files.zip › Supplementary_Figures_rev.pdf]

SUPPLEMENTARY DATA

Supplementary Figure S1

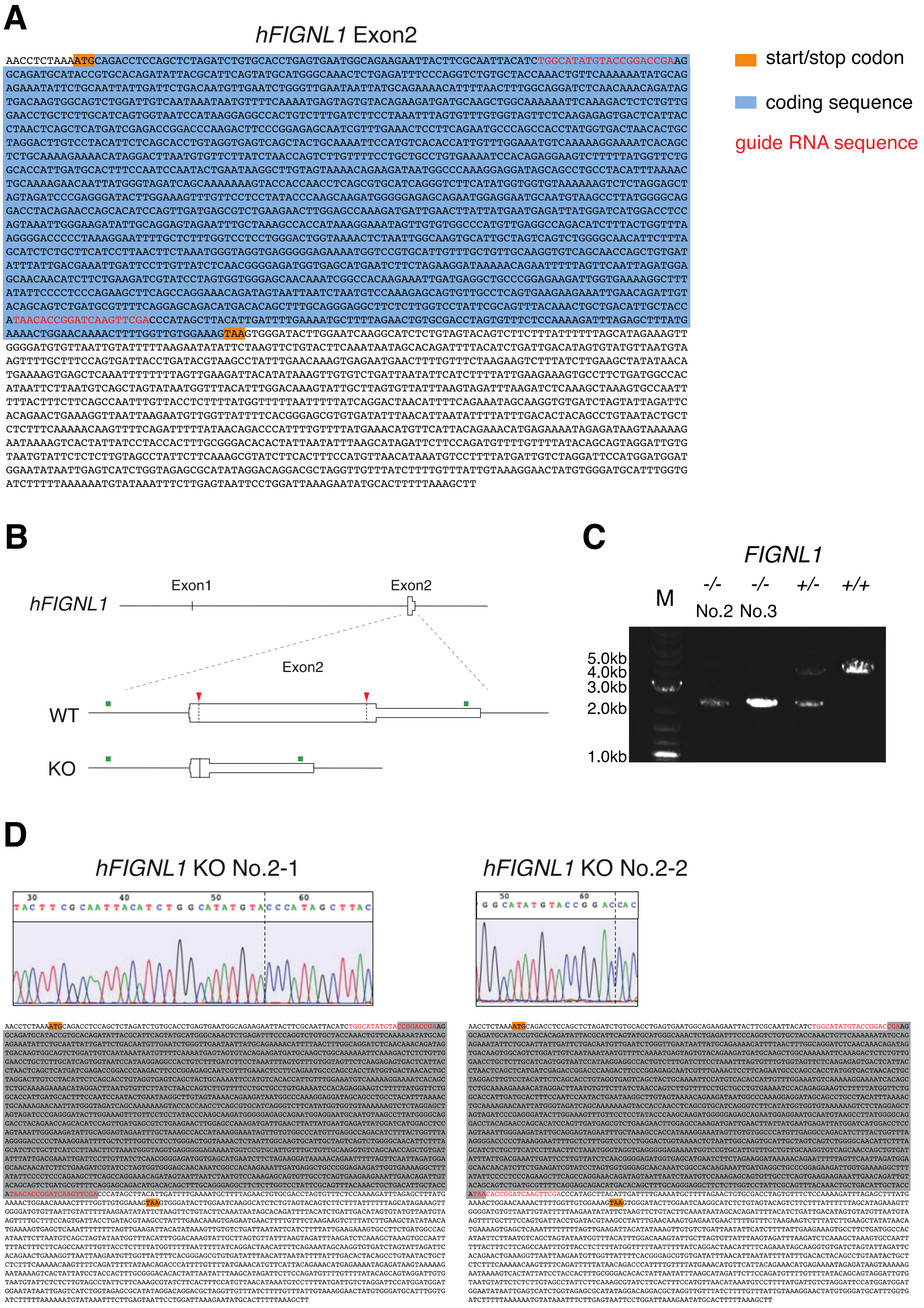

Supplementary Figure S1: Generation of human *FIGNL1* KO cell line

(A) DNA sequence of the exon2 of human *FIGNL1* gene. The start/stop codons and coding sequence are highlighted in orange and blue, respectively. Red letters represent Cas9/sgRNA target sites.

(B) Schematics of the human *FIGNL1* genomic locus. The genomic locus of human *FIGNL1* is shown to scale. Exons are represented by boxes (top). Enlarged schematic showing the second exon of human *FIGNL1* with the knockout construct. Arrowheads and green lines indicate the positions of the sgRNAs and genotyping primers, respectively.

(C) *FIGNL1* PCR genotyping. Wild-type and mutant genes are shown as 4.0 kb and 2.2 kb fragments, respectively.

(D) Genomic DNA sequences of two *FIGNL1* alleles in *FIGNL1* KO cell line (No.2). The start/stop codons and deleted regions are highlighted in orange and grey, respectively. Red letters represent Cas9/sgRNA target sites.

Supplementary Figure S2

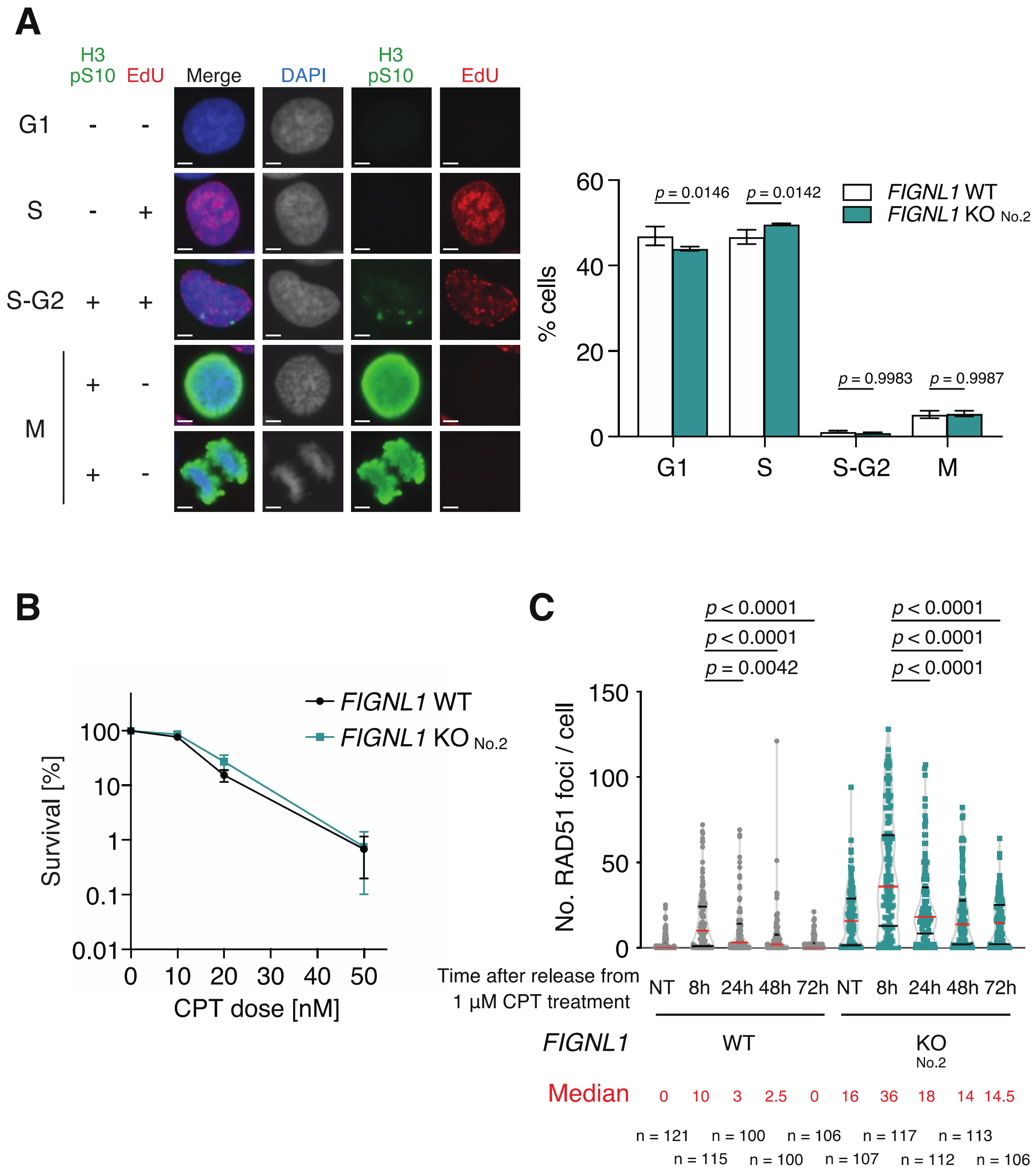

Supplementary Figure S2: Cell cycle distribution and CPT sensitivity of *FIGNL1* KO cells

(A) Incorporation of EdU and phosphorylation of H3 S10 (H3-pS10) in *FIGNL1* KO cells. The cells were incubated in media containing 10 μM EdU for 10 min, fixed, and stained with anti-PCNA antibody and an EdU labelling kit. Left, Representative images of H3-pS10 and EdU staining in control U2OS cells (WT) and *FIGNL1* KO cells (KO).

Scale bar = 5 μm. Right, Quantification of H3-pS10-positive and EdU-positive cells. More than 700 cells were counted for each sample. Data are presented as mean ± s.d. (n = 3, all biologically independent). Statistical significance was measured using an unpaired t-test.

(B) Clonogenic assay of control U2OS and *FIGNL1* KO cells. The cells were treated with the indicated concentrations of camptothecin (CPT) for 22 h. After 7 days, the number of colonies was counted. Relative numbers to the untreated control are shown. Data are presented as mean ± s.d. (n = 3, all biologically independent).

(C) Quantification of RAD51 foci in control U2OS cells and *FIGNL1* KO cells after CPT treatment. After the treatment with 1 μM CPT for 1 h, cells were washed and incubated in CPT-free media. The number of RAD51 foci per cell was counted at indicated time points. More than 100 cells were counted for each sample. Data are presented as median (red line) with IQR (black dashed line).

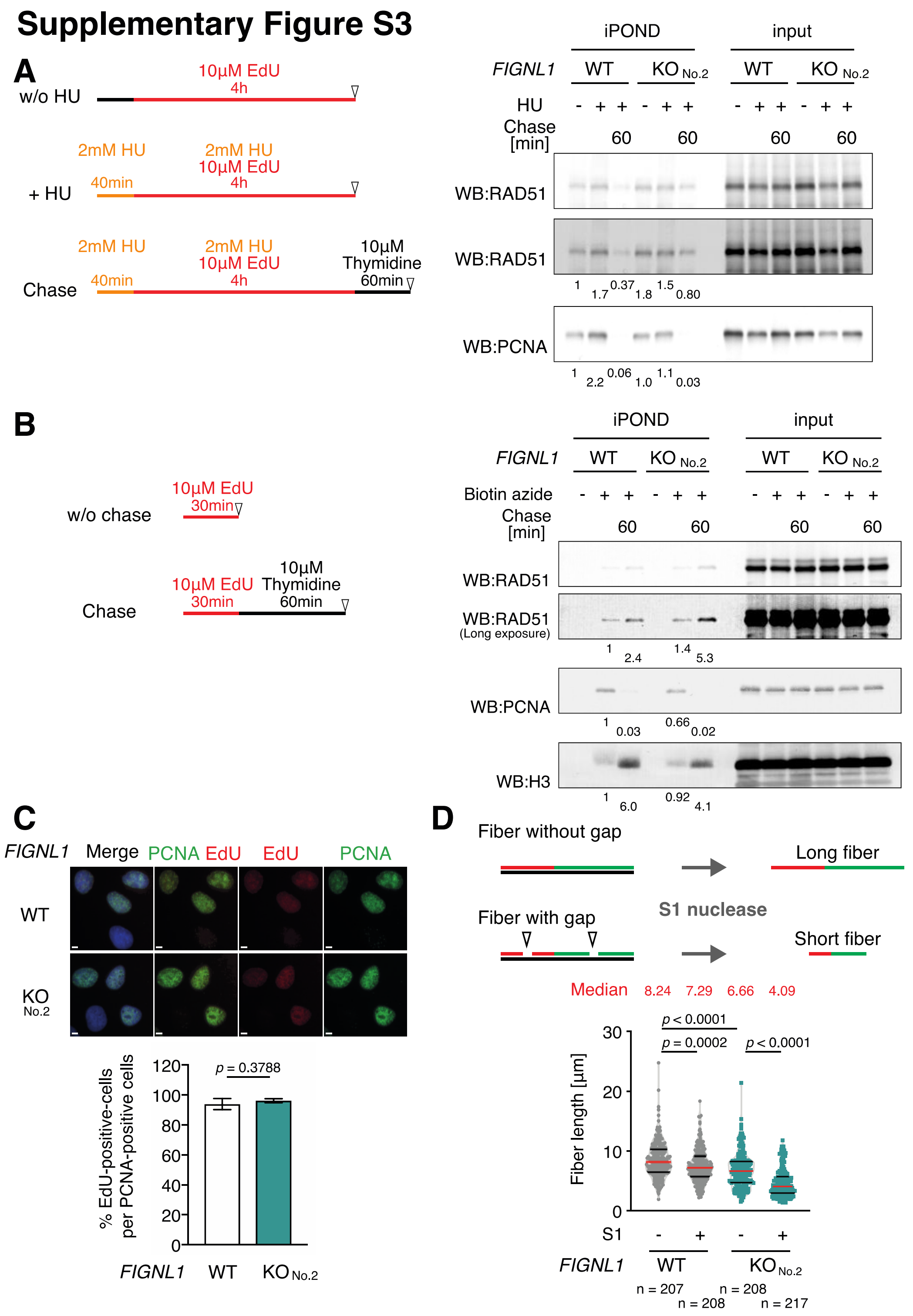

Supplementary Figure S3: Persistence of RAD51 after fork restart in *FIGNL1* KO cells

(A) Schematic of iPOND assay to quantify proteins associated with EdU-incorporated DNA strands under replication stress. U2OS cells were incubated in the presence of 2 mM HU and 10 μM EdU for 4 h (+HU), and chased with 10 μM thymidine for 60 min

(Chase). Untreated cells were incubated in the presence of 10 μM EdU for 4 h. Right, western blotting analysis of iPOND samples. Streptavidin pull-down samples and inputs were probed with the indicated antibodies. Relative band intensities to non-treated wild-type samples are shown below images.

(B) Schematic of iPOND assay to quantify proteins associated with EdU-incorporated DNA strands under unchallenged conditions. U2OS cells were incubated in the presence of 10 μM EdU for 30 min and chased with 10 μM thymidine for 60 min. Right, western blotting analysis of iPOND samples. Streptavidin pull-down samples and inputs were probed with the indicated antibodies. Relative band intensities to non-treated wild-type samples are shown below images.

(C) Incorporation of EdU after fork restart in *FIGNL1* KO cells. The cells were treated with 2 mM HU for 4 h. After washing, cells were incubated in media containing 10 μM EdU for 30 min and stained with anti-PCNA antibody and an EdU labelling kit. Left, Representative images of PCNA and EdU staining in control U2OS cells (WT) and *FIGNL1* KO cells (KO). Scale bar = 5 μm. Right, Quantification of PCNA focus-positive cells with EdU foci in the indicated cell lines. More than 200 cells were counted for each sample. Data are presented as mean ± s.d. (n = 3, all biologically independent).

(D) Top, Schematic for detection of ssDNA gap on newly synthesized DNA using S1 nuclease. Before stretching genomic DNA on glass slides, samples were treated with S1 nuclease to cleave DNA containing ssDNA gap. Bottom, Quantification of DNA fibre length with or without S1 nuclease treatment. More than 200 fibres were counted for each sample. Data are presented as median (red line) with IQR (black dashed line).

Supplementary Figure S4

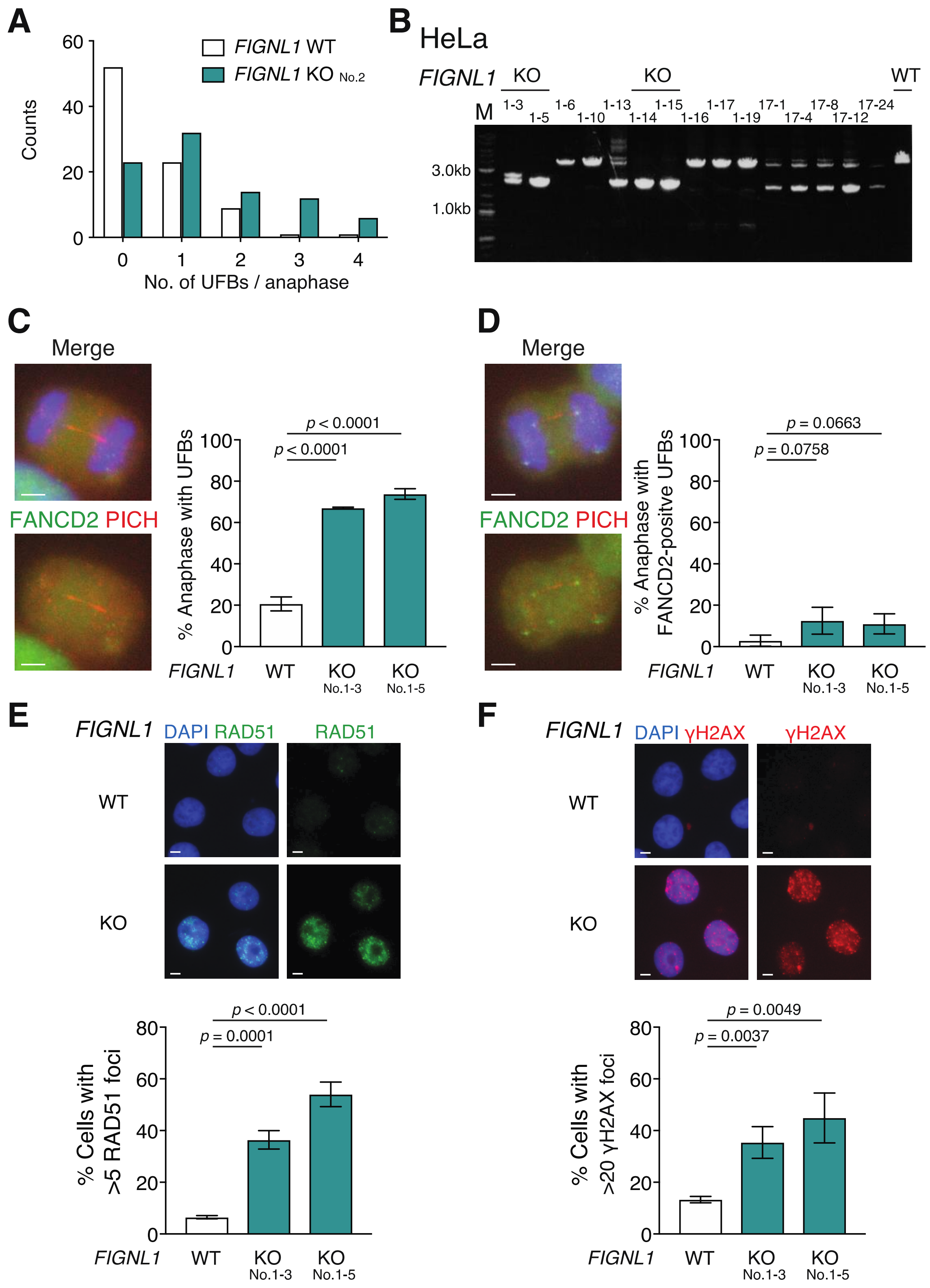

Supplementary Figure S4: UFB formation and RAD51 persistence in *FIGNL1* KO HeLa cells

(A) Anaphase cells in Figure. 3B were categorized based on the number of PICH-coated UFBs per cell.

(B) *FIGNL1* PCR genotyping in HeLa cells. Wild-type and mutant genes are shown as 4.0 kb and 2.2 kb fragments, respectively. Positions of genotyping primers and sgRNAs are shown in Supplementary Figure S1A, B.

(C) Left, Representative images of UFBs stained with PICH and FANCD2 antibodies. Scale bar = 5 μm. Right, Quantification of anaphase cells with PICH-coated UFBs in the indicated HeLa cell lines. More than 50 anaphase cells were counted for each sample. Data are presented as mean ± s.d. (n = 3, all biological independents).

(D) Left, Representative images of UFBs with FANCD2 foci at the extremity. Scale bar = 5 μm. Right, Quantification of anaphase cells with FANCD2-positive UFBs in the indicated HeLa cell lines. More than 50 anaphase cells were counted for each sample. Data are presented as mean ± s.d. (n = 3, all biological independents).

(E) Left, Representative images of immunofluorescence analysis of RAD51 foci in control U2OS (WT) and *FIGNL1* KO cells (KO). Scale bar = 5 μm. Right, Quantification of RAD51 focus-positive cells (> 5 foci/cell) in the indicated HeLa cell lines. More than 200 cells were counted for each sample. Data are presented as mean ± s.d. (n = 3, all biologically independent).

(F) Left, Representative images of immunofluorescence analysis of γH2AX foci in control U2OS (WT) and *FIGNL1* KO cells (KO). Scale bar = 5 μm. Right, Quantification of γH2AX focus-positive cells (> 20 foci/cell) in the indicated HeLa cell lines. More than 200 cells were counted for each sample. Data are presented as mean ± s.d. (n = 3, all biological independents).

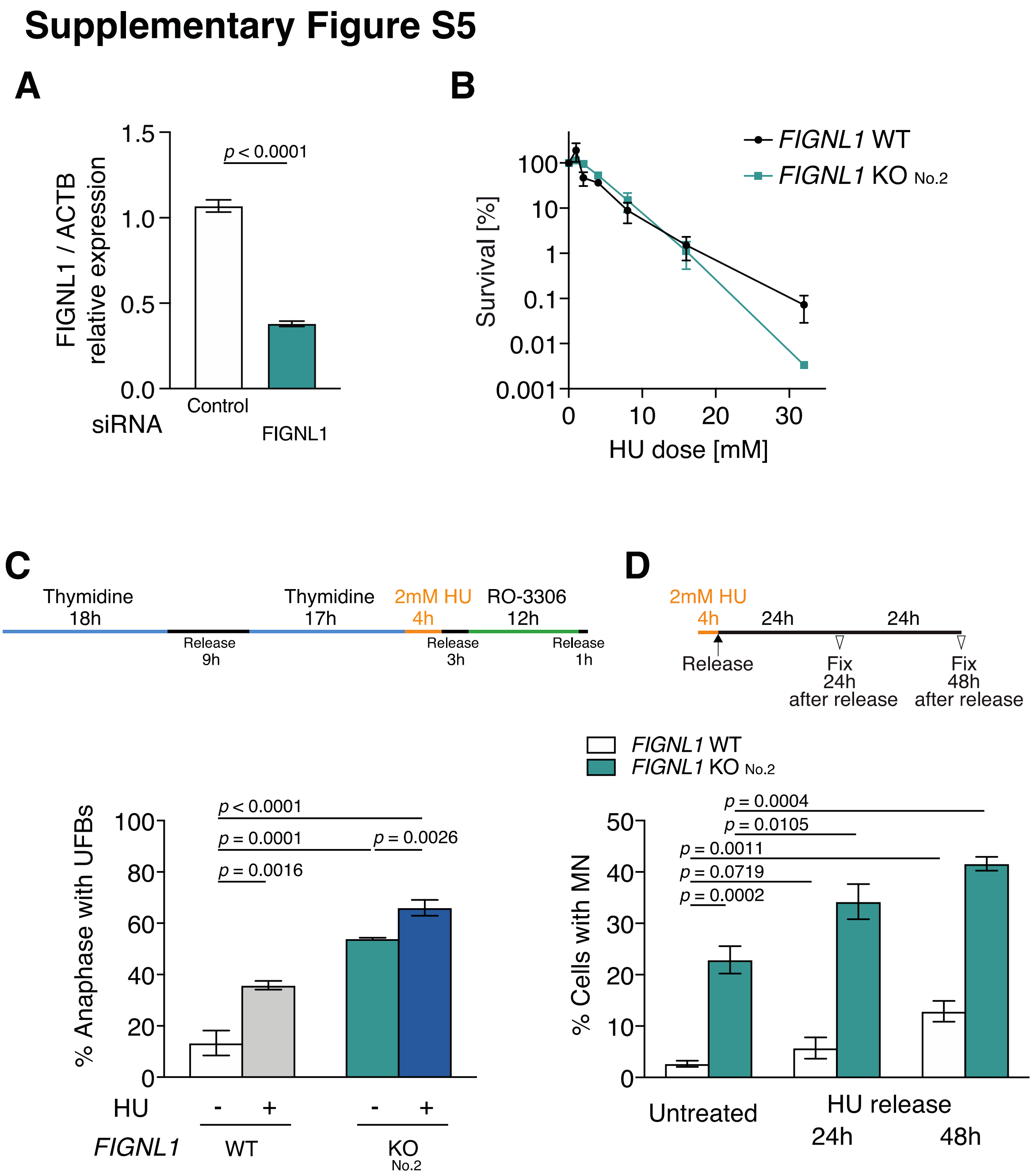

Supplementary Figure S5: Genome instability in *FIGNL1* KO cells is exacerbated by transient replication stress

(**A**) Fold reduction in *FIGNL1* mRNA in cells transfected with siRNA against human FIGNL1 and control. The expression of human FIGNL1 mRNA was assessed by RT-

qPCR and normalized by the expression of ACTB. Data are presented as mean ± s.d. (n=3, all biological independents).

(B) Relative survival of control U2OS cells and *FIGNL1* KO cells transiently exposed to the indicated concentrations of HU. Cells incubated in the presence of the indicated concentration of HU for 24 h. After the release from HU treatment, the cells were incubated for 7 days and subjected to a cell titer Glo assay. Relative luminescence to untreated samples is shown. Data are presented as mean ± s.d. (n = 3, all biological independents).

(C) Top, schematic to analyse UFB formation after release from HU treatment. Bottom: Quantification of anaphase cells with PICH-coated UFBs in the indicated cell lines. After a double thymidine block, the cells were treated with 2 mM HU for 4 h. After the release from HU treatment, the cells were fixed at the indicated points. More than 50 anaphase cells were counted in each sample. Data are presented as mean ± s.d. (n = 3, all biologically independent).

(D) Top, schematic to analyse micronuclei formation after release from transient HU treatment. Control U2OS cells and *FIGNL1* KO cells were treated with 2 mM HU for 4 h and released into HU-free media. At 24 and 48 h after release, the cells were fixed and stained with DAPI. Bottom, Quantification of cells containing micronuclei in the indicated cell lines. More than 200 cells were counted for each sample. Data are presented as mean ± s.d. (n = 3, all biologically independent).

Supplementary Figure S6

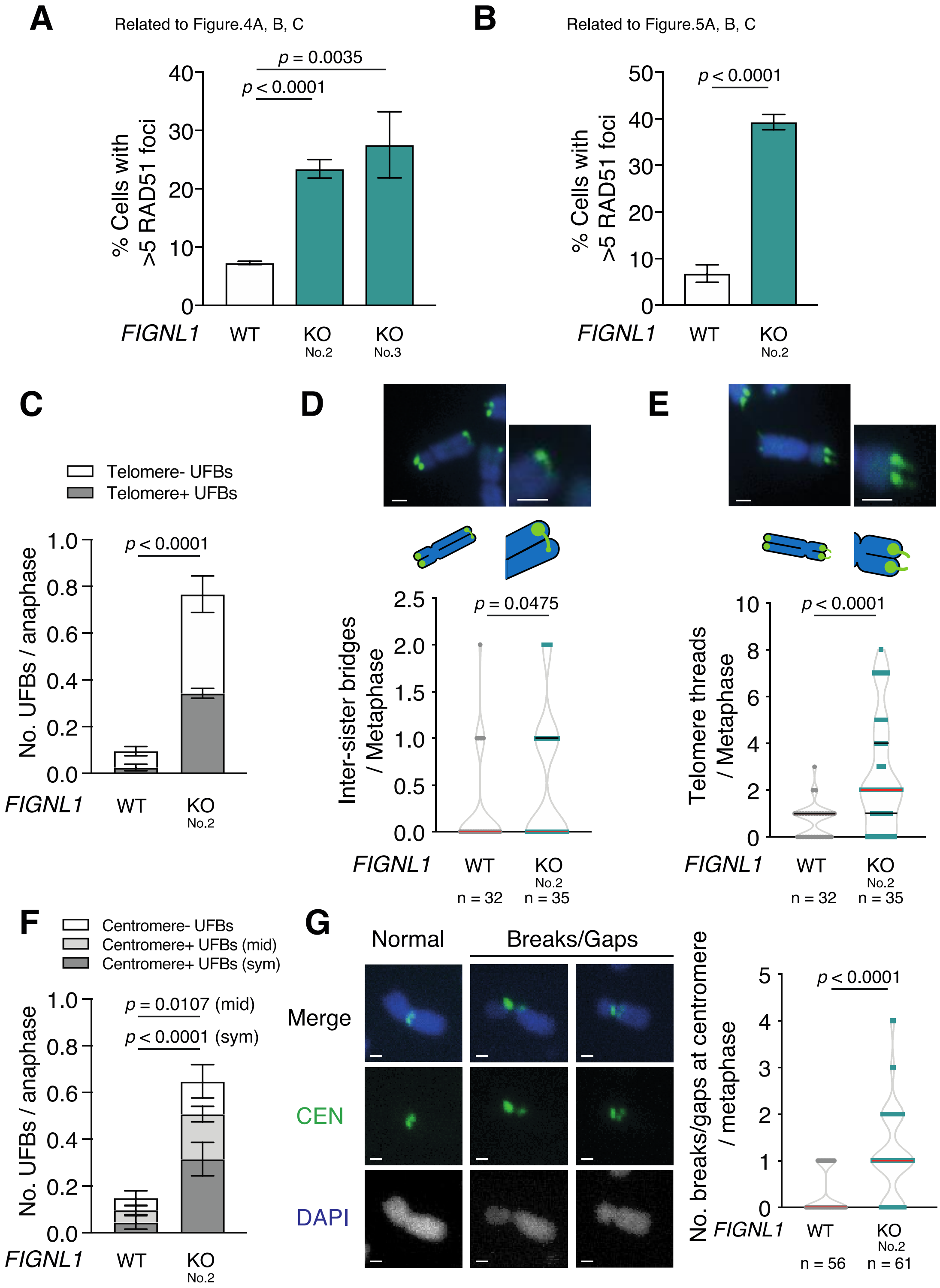

Supplementary Figure S6: Genome instability at centromere and telomere in *FIGNL1* KO cells

(A)-(B) Immunofluorescence analysis of RAD51 focus in control U2OS cells and *FIGNLl1* KO cells. Quantification of RAD51 focus-positive cells (> 5 foci/cell) in the indicated cell lines (A: related to Figure 4A, B, C; and B: related to Figure 5A, B, C). More than 200 cells were counted for each sample. Data are presented as mean ± s.d. (n = 3, all biologically independent).

(C) Quantification of the number of UFBs per cell in the indicated cell lines. UFBs were classified as UFBs with or without telomere signal on the bridge (telomere+ UFBs or telomere- UFBs, respectively). Data are presented as mean ± s.d. (n = 3, all biologically independent).

(D)-(E) Top, Schematic and representative images of metaphase chromosome with telomere bridge (C) and thread-like telomere signal (D). Scale bar = 2 μm. Bottom, quantification of telomere bridges (C) and thread-like telomeres (D) per metaphase in the indicated cell lines. Data are presented as median (red line) with IQR (black dashed line).

(F) Quantification of the number of UFBs per cell in the indicated cell lines. UFBs were classified as UFBs with centromere signal in the middle region of the bridge (centromere+ UFBs (mid)), UFBs with centromere signals at the terminus of the bridge (centromere+ UFBs (sym)), or UFBs without centromere signal on the bridge (centromere- UFBs). Data are presented as mean ± s.d. (n = 3, all biologically independent).

(G) Left, Representative image of a metaphase chromosome with breakage at centromere. Metaphase spreads were hybridized with centromere FISH probe. Scale bar = 1 μm. Right Quantification of chromosomes with breakage at centromere per metaphase in the indicated cell lines. Chromosomes with split centromere FISH

signals were counted. Data are presented as median (red line) with IQR (black dashed line).

Supplementary Figure S7

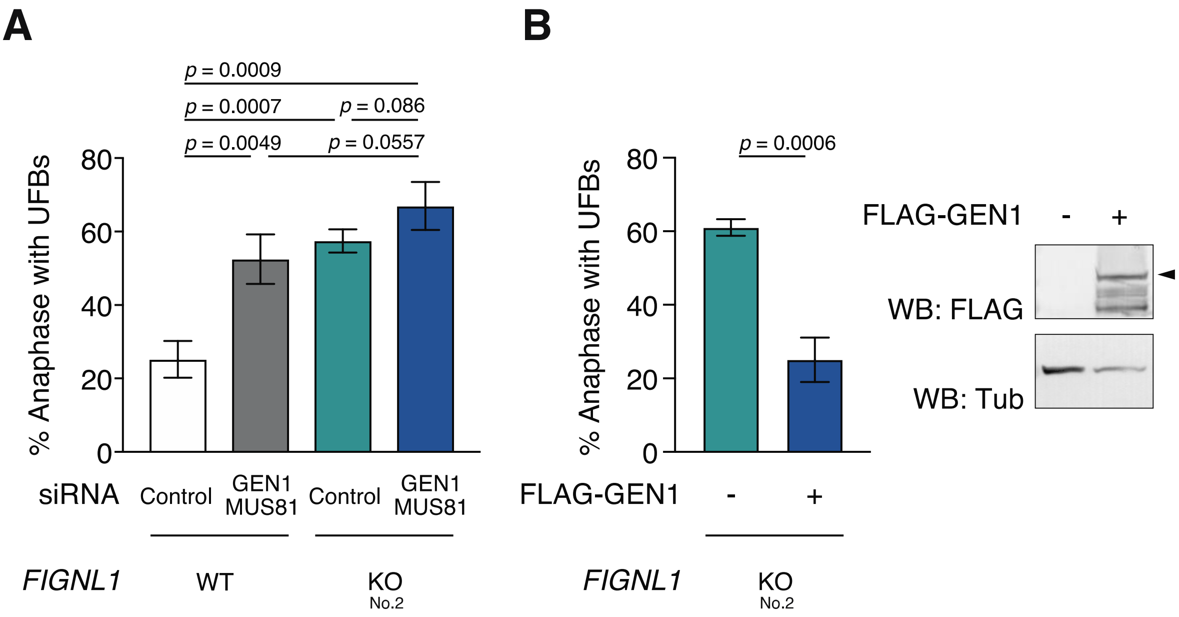

Supplementary Figure S7: FIGNL1 and resolvases are epistatic in terms of suppressing UFB formation

(A) Quantification of anaphase cells with PICH-coated UFBs in the indicated cell lines. More than 50 anaphase cells were counted for each sample. Data are presented as mean ± s.d. (n = 3, all biologically independent).

(B) Left, Quantification of anaphase cells with PICH-coated UFBs in *FIGNL1* KO cells with or without the exogenous expression of FLAG-GEN1. More than 50 anaphase cells were counted for each sample. Data are presented as mean ± s.d. (n = 3, all biologically independent). Right, Western blotting analysis of FLAG-GEN1-expressing cells. The WCEs were probed with the indicated antibodies. Arrowhead indicates FLAG-GEN1 protein.

Supplementary Figure S8

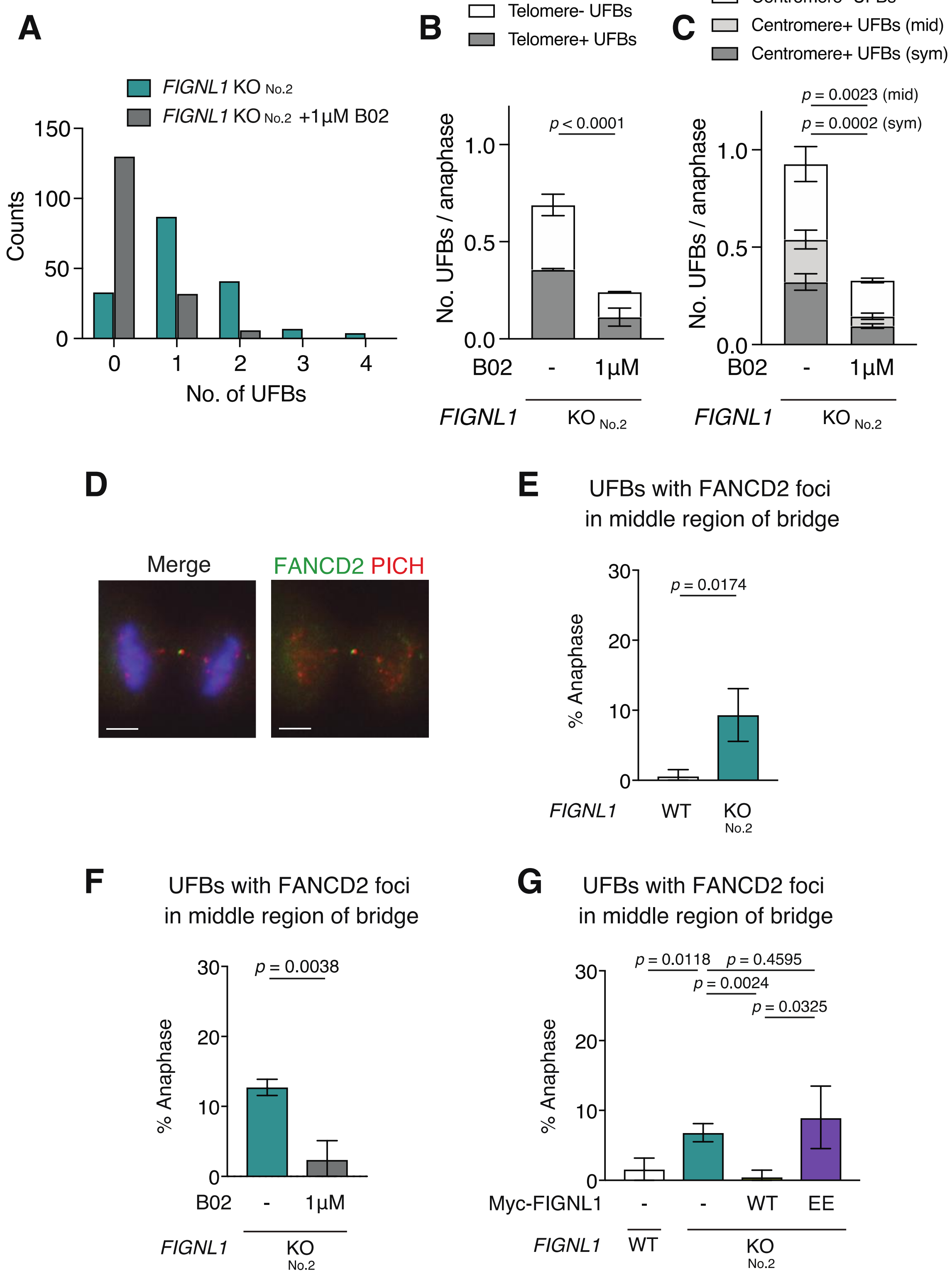

Supplementary Figure S8: The features of UFBs in *FIGNL1* KO cells

(A) Anaphase cells in Figure. 6C were categorized based on the number of PICH-coated UFBs per cell.

(B) Quantification of the number of UFBs per cell in the indicated cell lines. UFBs were classified as UFBs with or without telomere signal on the bridge (telomere+ UFBs or telomere- UFBs, respectively). Data are presented as mean ± s.d. (n = 3, all biologically independent).

(C) Quantification of the number of UFBs per cell in the indicated cell lines. UFBs were classified as UFBs with centromere signal in the middle region of the bridge (centromere+ UFBs (mid)), UFBs with centromere signals at the terminus of the bridge (centromere+ UFBs (sym)), or UFBs without centromere signal on the bridge (centromere- UFBs). Data are presented as mean ± s.d. (n = 3, all biologically independent).

(D) Representative images of UFBs with FANCD2 focus in the middle region of the bridge.

(E)-(G) Quantification of anaphase cells with UFBs containing FANCD2 foci in the middle region of the bridge in *FIGNL1* KO cells (B), B02-treated U2OS cells (C), and *FIGNL1* KO cells complemented with Myc-FIGNL1 or Myc-FIGNL1-EE (D). More than 50 anaphase cells were counted for each sample. Data are presented as mean ± s.d. (n = 3, all biologically independent). Scale bar = 5 μm.

Supplementary Figure S9

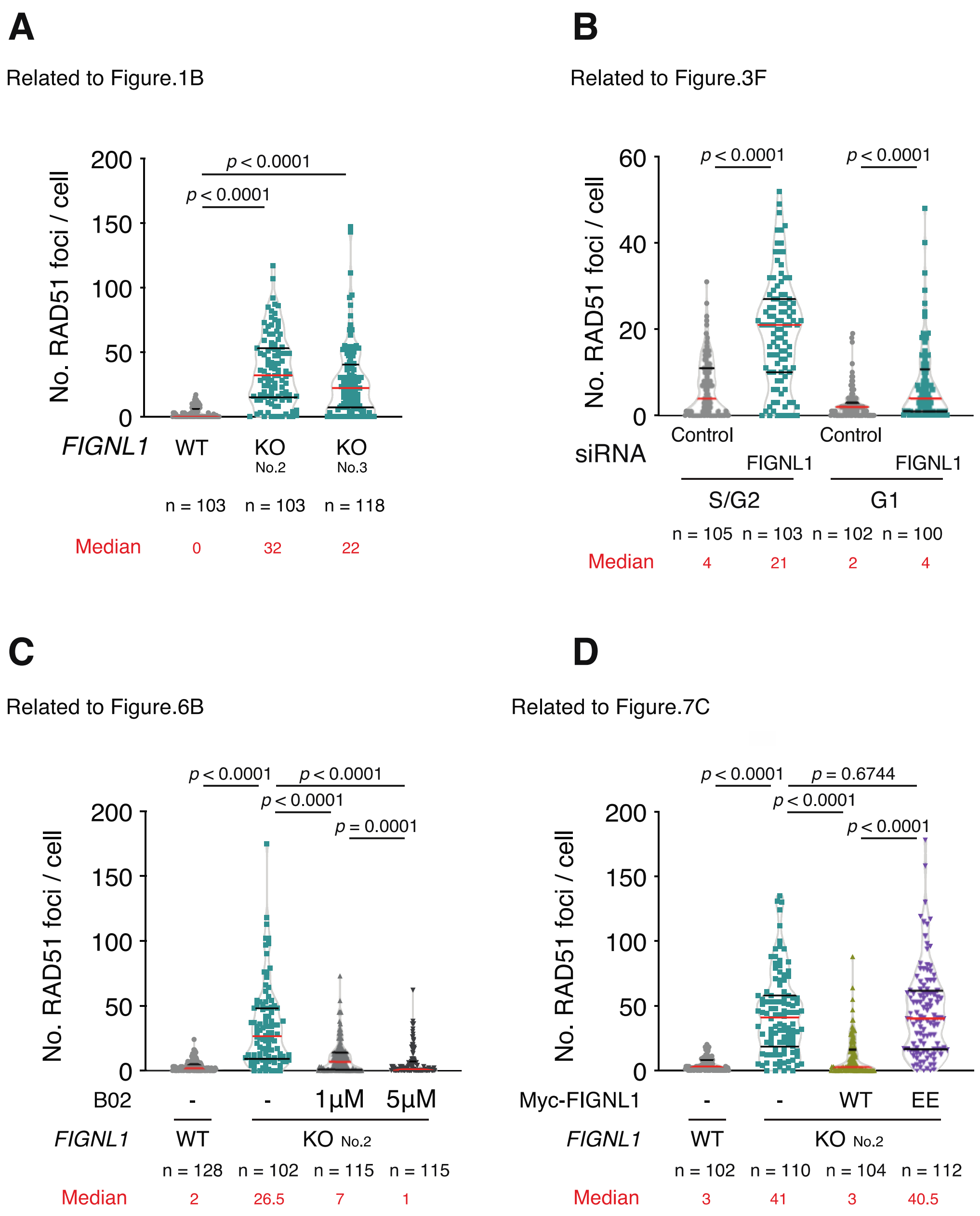

Supplementary Figure S9: The number of RAD51 foci in *FIGNL1*-deficient cells

(A) Quantification of RAD51 foci in control U2OS cells and *FIGNL1* KO cells (related to Figure 1B). The number of RAD51 foci per cell was counted. More than 100 cells were analysed for each sample. Data are presented as median (red line) with IQR (black dashed line).

(B) Quantification of RAD51 foci in U2OS cells treated with siRNA against human *FIGNL1* and control (related to Figure 3F left). The number of RAD51 foci per cell was

counted. More than 100 cells were analysed for each sample. Data are presented as median (red line) with IQR (black dashed line).

(C) Quantification of RAD51 foci in control U2OS cells and *FIGNL1* KO cells treated with the indicated concentration of B02 (related to Figure 6B). The number of RAD51 foci per cell was counted. More than 100 cells were analysed for each sample. Data are presented as median (red line) with IQR (black dashed line).

(D) Quantification of RAD51 foci in control U2OS cells and *FIGNL1* KO cells with or without expression of Myc-FIGNL1 mutants (related to Figure 7C). The number of RAD51 foci per cell was counted. More than 100 cells were analysed for each sample. Data are presented as median (red line) with IQR (black dashed line)

Supplementary Figure S10

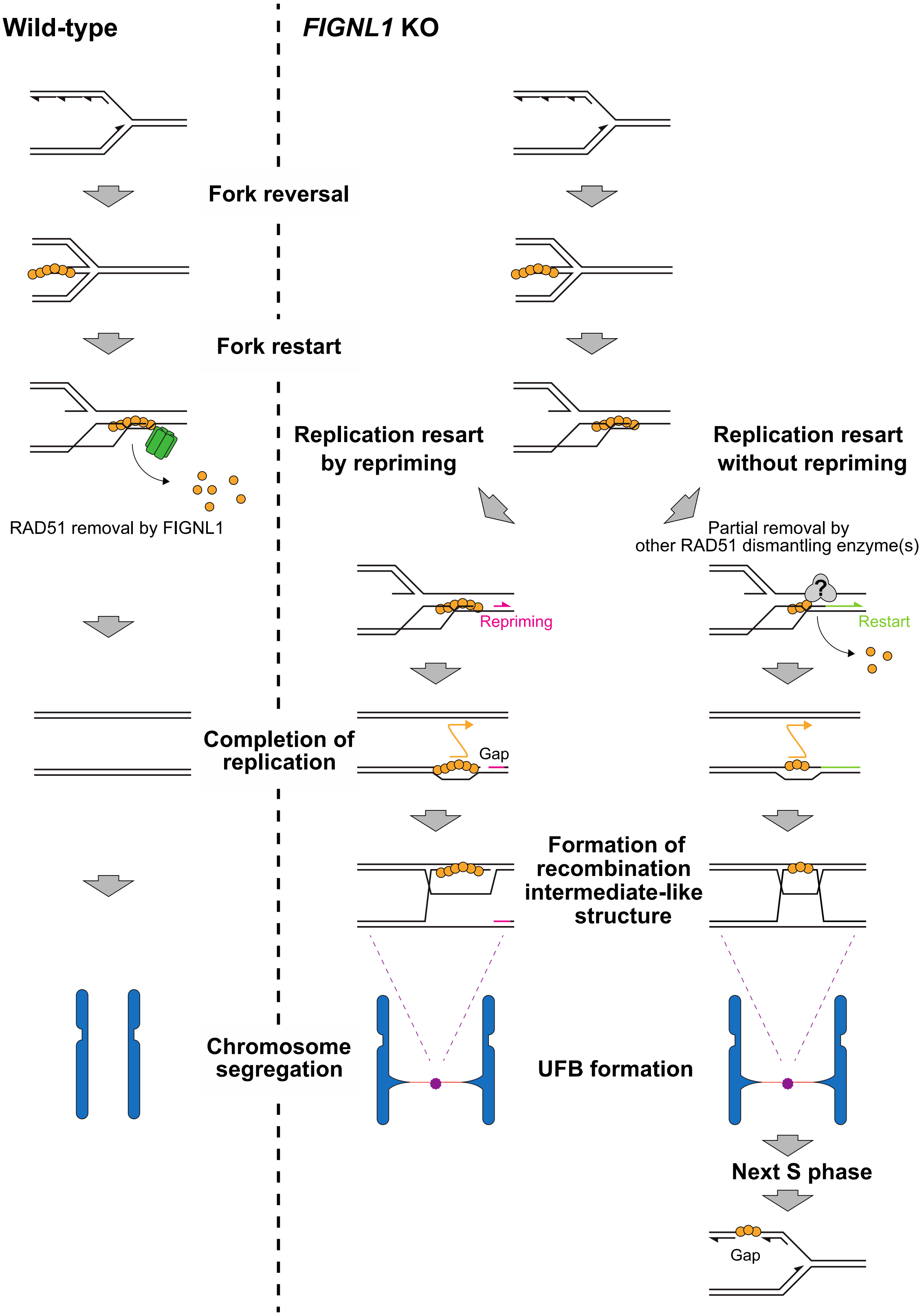

Supplementary Figure S10: Model showing the disassembly of RAD51 by FIGNL1 and RAD51-mediated UFB formation in the absence of FIGNL1

Supplementary Figure S11

Related to Supplementary Figure.1C

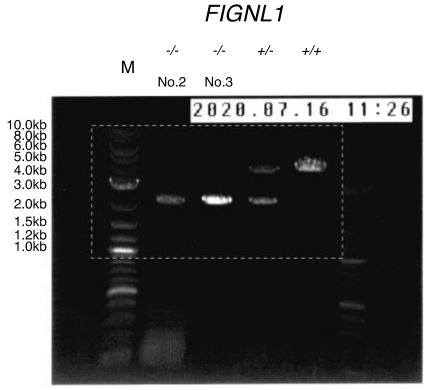

Related to Supplemantary Figure.4B

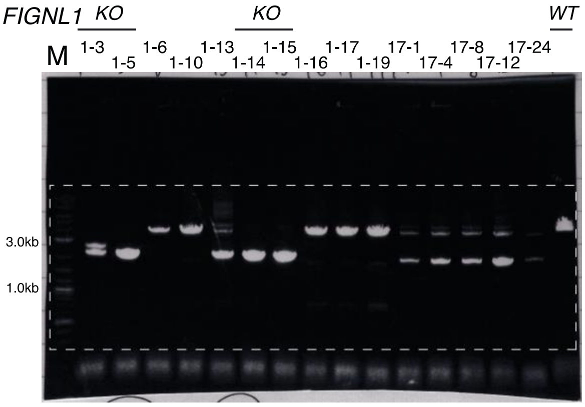

Related to Figure.2B

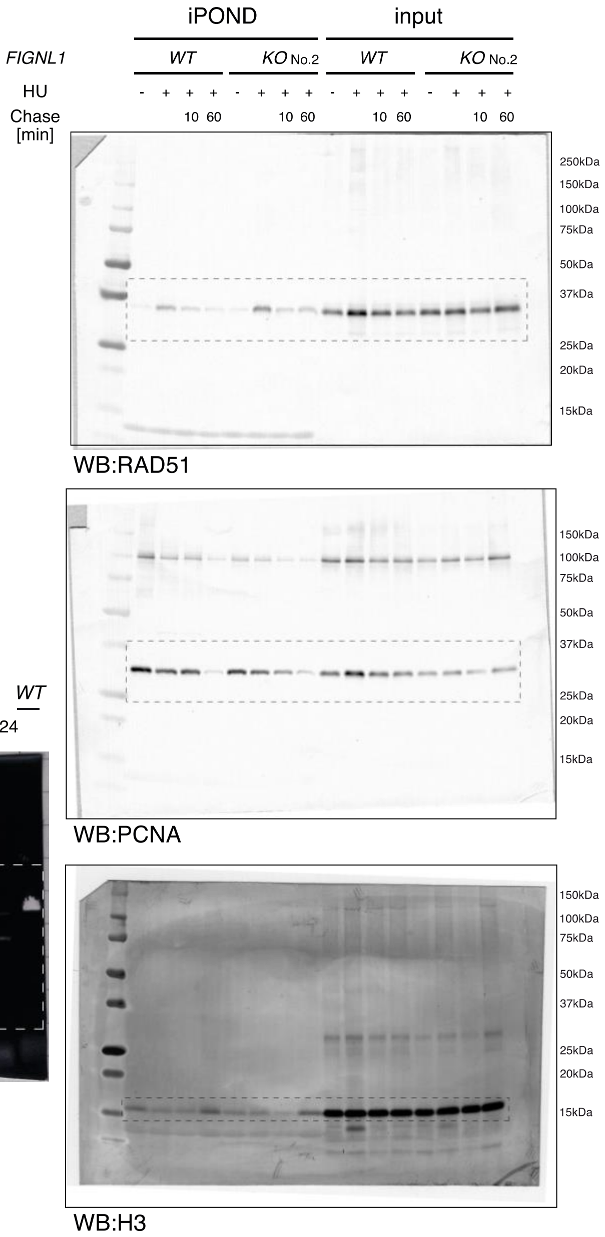

Supplementary Figure S11

Related to Figure.7A

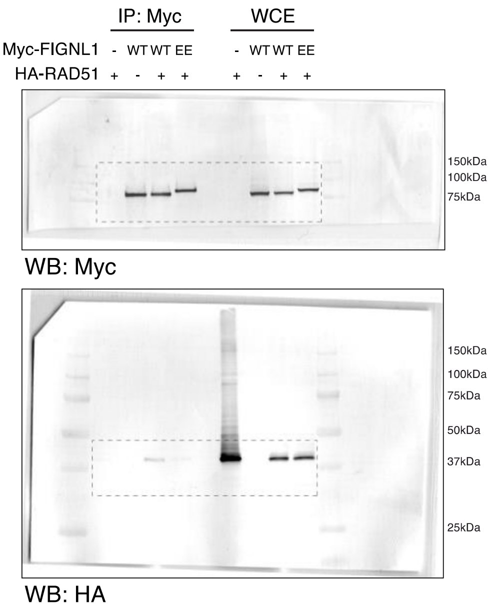

Related to Figure.7B

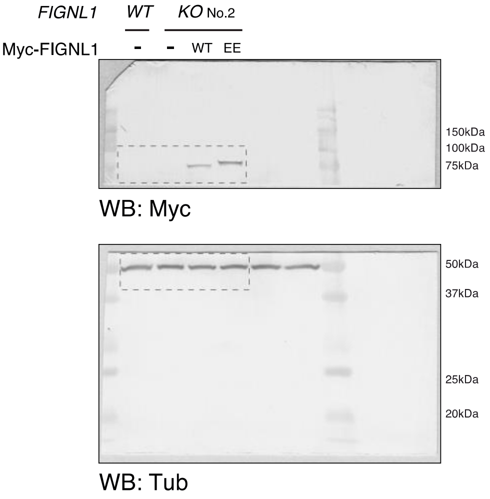

Related to Figure.7D

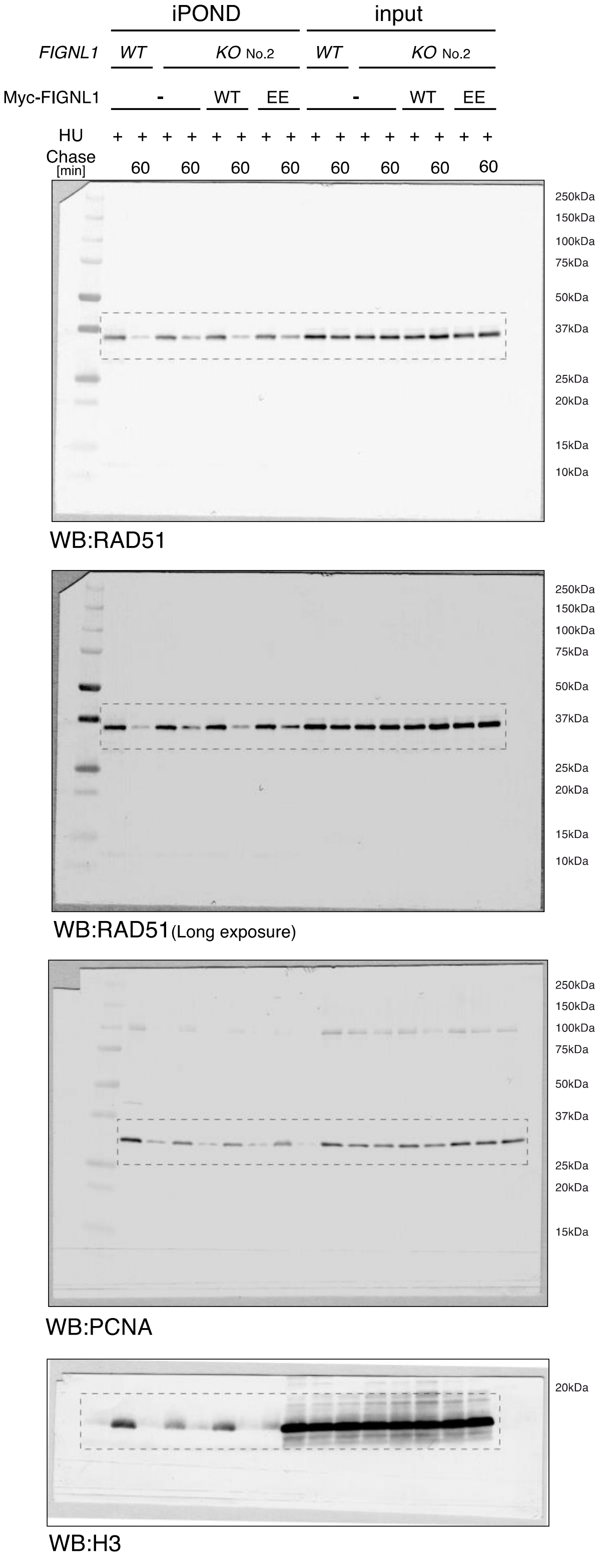

Supplementary Figure S11

Related to Supplementary Figure.3A

Related to Supplementary Figure.3B

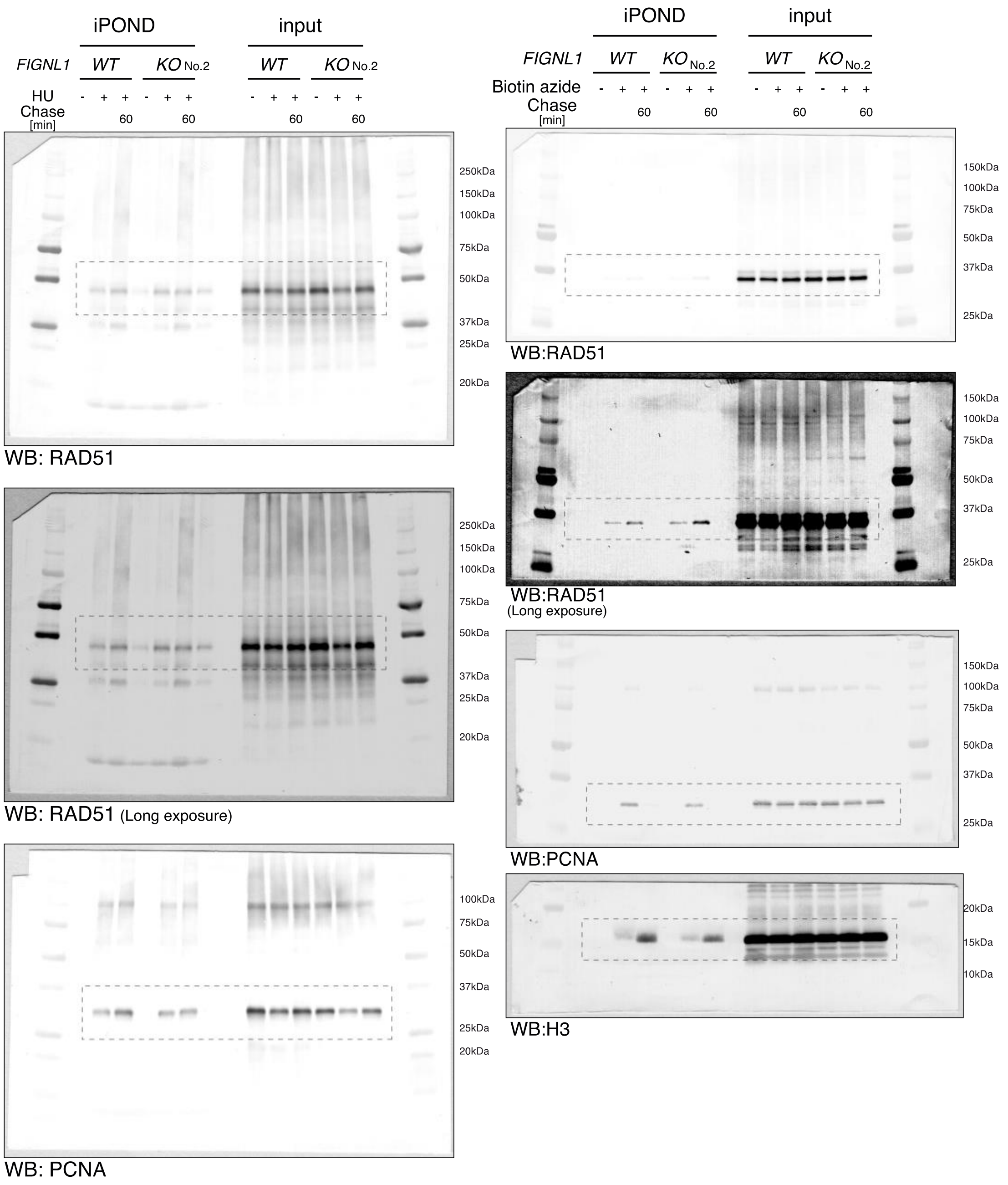

Supplementary Figure S11: Uncropped scans for western blots and agarose gel
